# Supplementary material for: Long-term comparison between index selection and optimal independent culling in plant breeding programs with genomic prediction
Source: PLoS One. 2021 May 10;16(5):e0235554. doi: 10.1371/journal.pone.0235554 (PMC8109766; doi:10.1371/journal.pone.0235554)
Supplement: S1 File — (DOCX) [file pone.0235554.s001.docx]

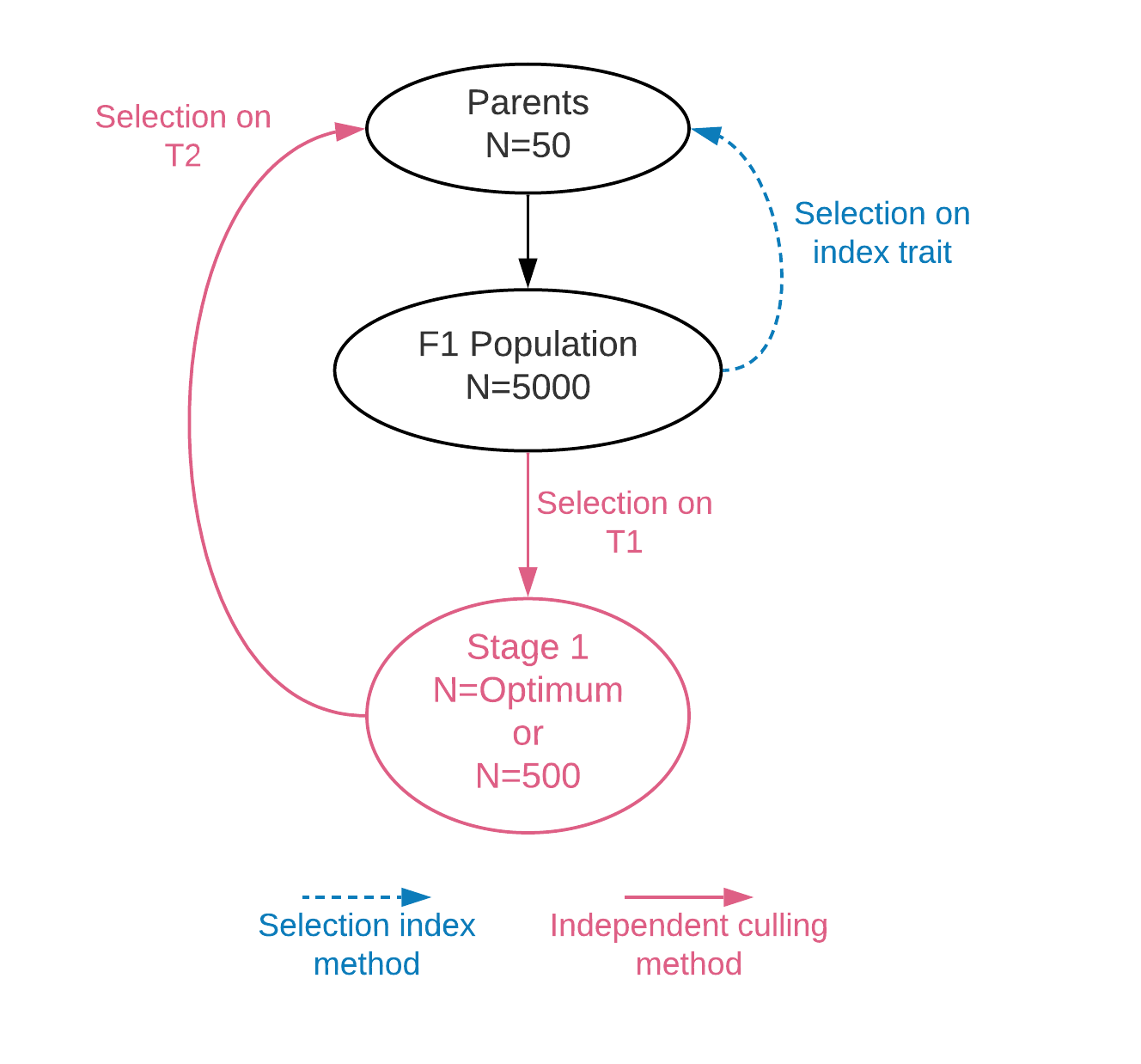


S1 Fig. Scheme for selection of parents using an economic selection index or independent culling with two traits (T1 and T2) under selection. The index trait is the sum of the estimated breeding values of T1 and T2 weighted by their economic importance. Number of individuals (N) is shown for each selection stage. The optimum value corresponds to the selected proportion that maximizes net genetic gain in each cycle of selection.


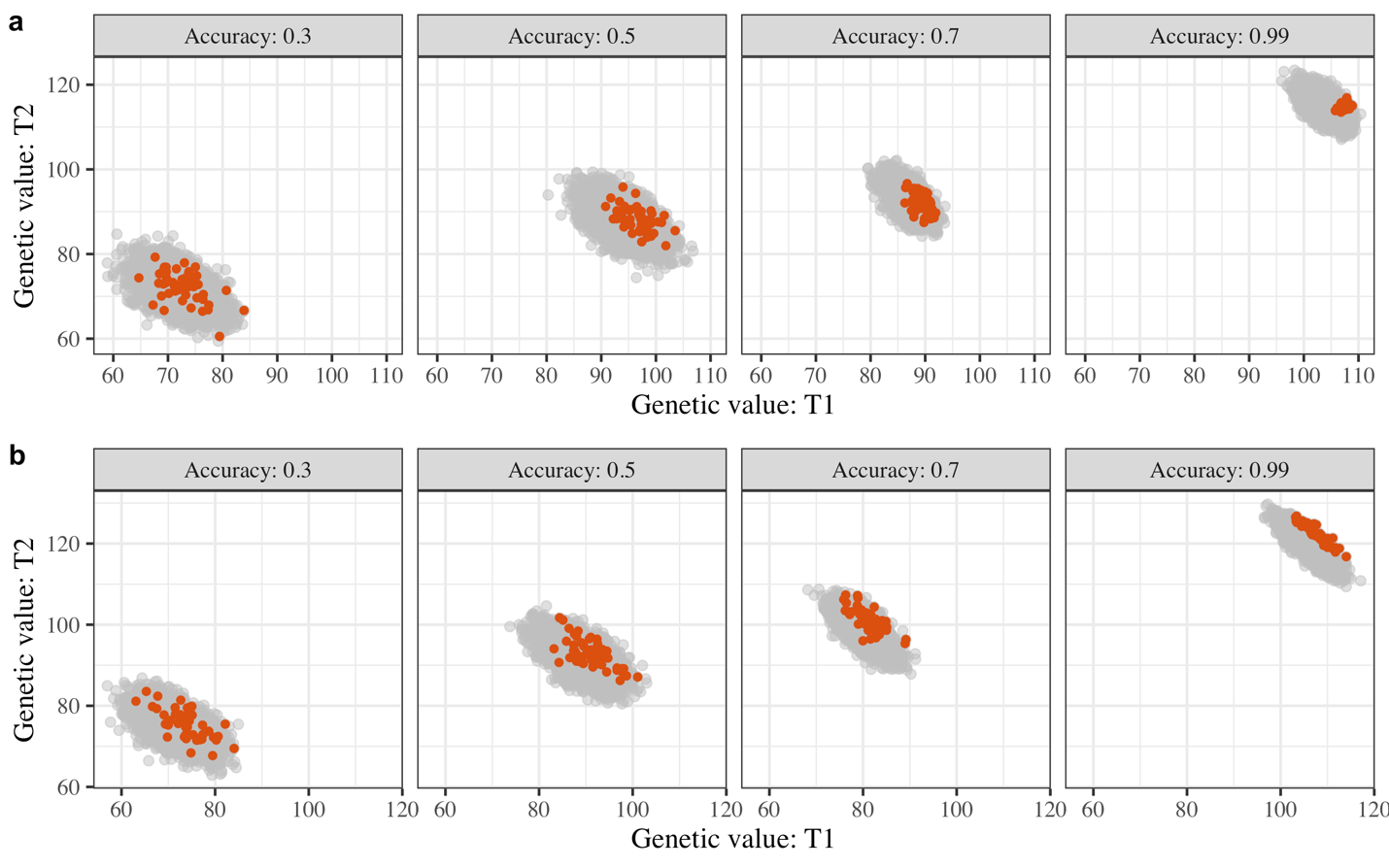


S2 Fig. Scatterplots of true genetic values for Trait 1 (T1) and Trait 2 (T2) of the genotypes in the F1 population (grey) and genotypes selected as parents (orange) in the 20^th^ cycle of selection using either independent culling (a) or a selection index (b) with different levels of accuracy, unfavourable genetic correlation between traits (-0.5), and Trait 2 relative economic importance of 1.0


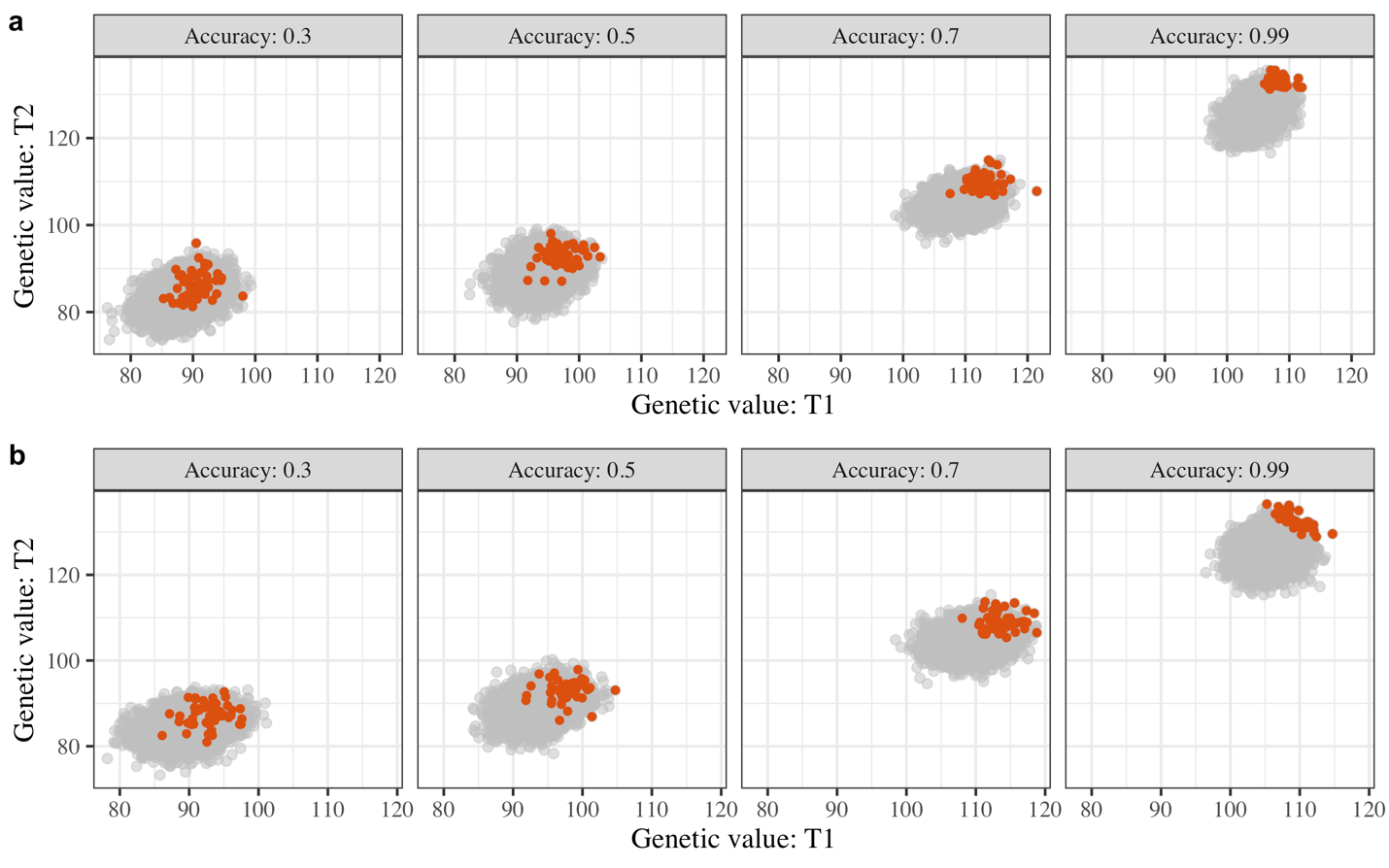


S3 Fig. Scatterplots of true genetic values for Trait 1 (T1) and Trait 2 (T2) of the genotypes in the F1 population (grey) and genotypes selected as parents (orange) in the third cycle of selection using either independent culling (a) or a selection index (b) with different levels of accuracy, favourable genetic correlation between traits (0.5), and Trait 2 relative economic importance of 1.0


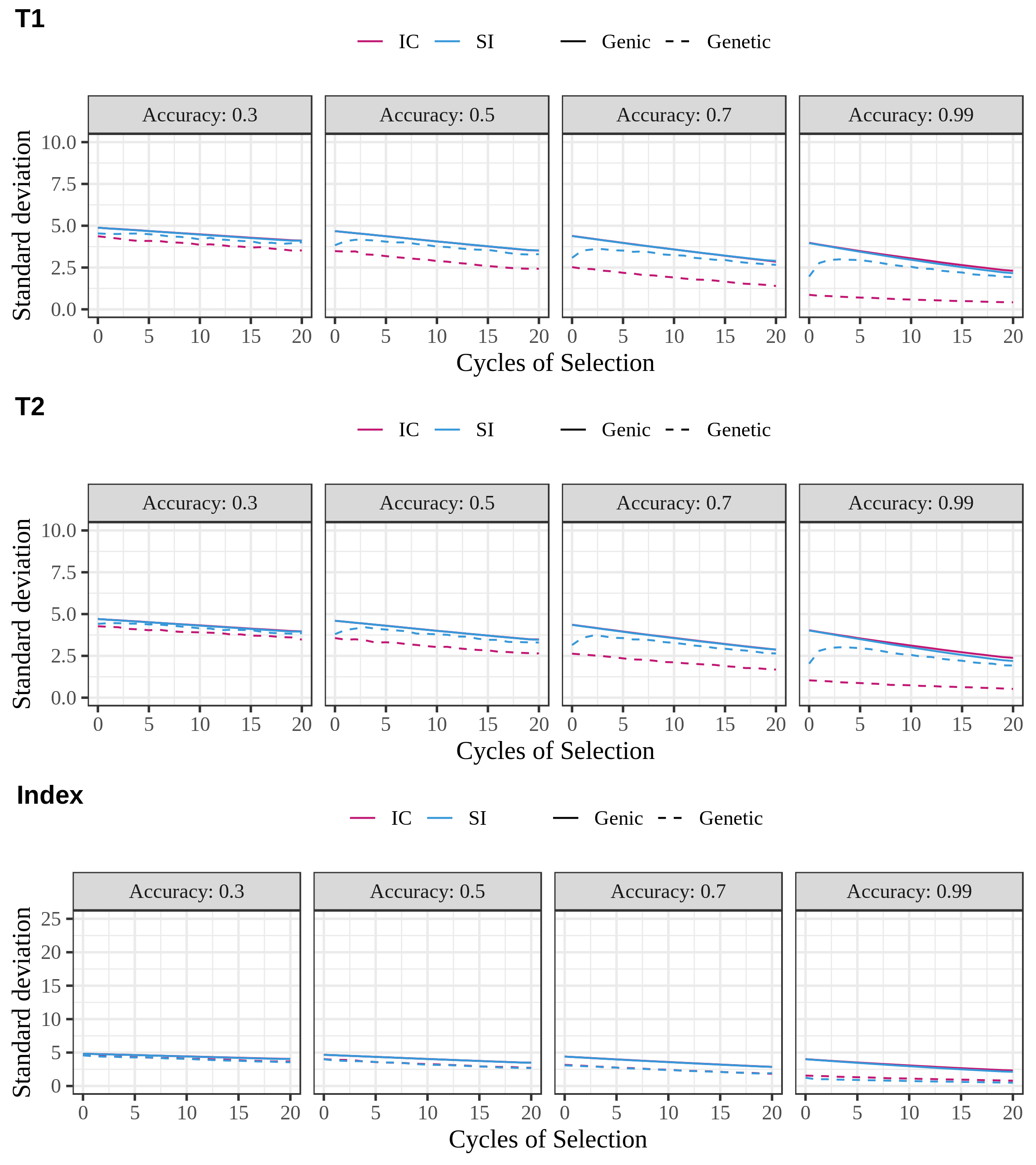


S4 Fig. Change in genic and genetic standard deviation for Trait 1 (T1), Trait 2 (T2) and Index Trait (Index) over 20 cycles of selection using either independent culling (IC) or a selection index (SI) with different levels of accuracy, proportion selected of 10%, unfavourably correlated traits, and T2 relative economic importance of 1.0

S1 Table. Values of mean proportion selected for T1 used to achieve optimal culling levels over the 20 cycles of selection under three levels of relative economic importance (REI). Traits are unfavourably correlated (-0.5).

| Cycle | Mean Proportion Selected (%) | | |
| --- | --- | --- | --- |
|  | REI: 1.0 | REI: 2.5 | REI: 5.0 |
| 0 | 27 | 95 | 100 |
| 1 | 26 | 95 | 100 |
| 2 | 27 | 95 | 100 |
| 3 | 27 | 95 | 99 |
| 4 | 28 | 95 | 99 |
| 5 | 29 | 95 | 99 |
| 6 | 28 | 94 | 99 |
| 7 | 27 | 94 | 99 |
| 8 | 28 | 95 | 99 |
| 9 | 29 | 94 | 99 |
| 10 | 30 | 94 | 98 |
| 11 | 29 | 93 | 99 |
| 12 | 30 | 93 | 99 |
| 13 | 31 | 92 | 99 |
| 14 | 30 | 91 | 98 |
| 15 | 30 | 91 | 99 |
| 16 | 30 | 92 | 98 |
| 17 | 30 | 91 | 98 |
| 18 | 31 | 90 | 98 |
| 19 | 30 | 89 | 99 |
| 20 | 31 | 90 | 98 |
| Average: | 28.95 | 93.00 | 98.86 |
