# Supplementary material for: Long-term comparison between index selection and optimal independent culling in plant breeding programs with genomic prediction
Source: PLoS One. 2021 May 10;16(5):e0235554. doi: 10.1371/journal.pone.0235554 (PMC8109766; doi:10.1371/journal.pone.0235554)
Supplement: S2 File — (PDF) [file pone.0235554.s002.pdf]

Code example: scenario one

```
library(AlphaSimR)

################################################################################
### Setting up parameters ###
################################################################################

# Economic weights
w1 = c(1,1)
w2 = c(1,2.5)
w3 = c(1,5.0)

#Initial parents mean, variance and genetic covariance between traits

# The values for each component trait must be provided and
# an extra value needs to be present for the function addIndexTrait.
# Also, an extra row and column on the correlation matrices must be present.
# The chosen extra values will be replaced by the function addIndexTrait.
# Correlation matrices must be positive definite.

initMeanG = c(50 ,50, 50)
initVarG = c(50, 50, 50)


corA = matrix(c(1    ,-0.5, 0,
                -0.5,    1, 0,
                0    ,    0, 1), nc=3, byrow=T)

corDD = matrix(c(1, 0, 0,
                 0, 1, 0,
                 0, 0, 1), nc=3, byrow=T)

#Number of entries and parents

nParents = 50
nPop = 5000

#Relates to error variance for an entry mean
varE = c(700,700,700)

#Number of QTL per chromosome
nQtl = 1000
```

```r
###############################################################################
### Setting up functions ###
###############################################################################

#Function to get the EBVs

getEBV <- function(pop, accuracy, weights){
  ebv = pop@gv[,1:(ncol(pop@gv)-1)]
  #Target level of accuracy
  for(i in 1:(ncol(pop@gv)-1)){
    gv <- ebv[,i]
    sd <- sqrt(var(gv)* (1 - accuracy^2)/accuracy^2)
    error <- rnorm(sd = sd, mean = 0, n = length(gv))
    tmp <- gv + error

    ebv[,i] <- tmp
  }
  #getting G and P matrices
  G = cor(pop@gv)[1:(ncol(pop@gv)-1),1:(ncol(pop@gv)-1)]
  P = cor(ebv)
  #getting ebvs of component traits for Smith and Hazel index and saving on @ebv slot
  ebv = ebv%*%solve(P)%*%G
  #Creating index column
  Ind = rowSums(sweep(ebv,2,weights,"*"))
  pop@ebv <- cbind(ebv, Ind)

  return(pop)
}

#Function to add an index trait (pop@gv will be used to track changes on the index trait)

addIndexTrait = function(weights,simParam=NULL){
  if(is.null(simParam)){
    simParam = get("SP",envir=.GlobalEnv)
  }
  nTraits = length(weights)
  nLoci = simParam$traits[[1]]@nLoci

  addEff = numeric(nLoci)
  domEff = numeric(nLoci)
  intercept = numeric(1)
  for(i in 1:nTraits){
    addEff = addEff + simParam$traits[[i]]@addEff*weights[i]
    domEff = domEff + simParam$traits[[i]]@domEff*weights[i]
    intercept = intercept + simParam$traits[[i]]@intercept*weights[i]
  }
  trait = new("TraitAD",
              nLoci=nLoci,
              lociPerChr=simParam$traits[[1]]@lociPerChr,
              lociLoc=simParam$traits[[1]]@lociLoc,
```

```
              addEff=addEff,
              domEff=domEff,
              intercept=intercept)
  simParam$switchTrait(trait,nTraits+1L)
  invisible(NULL)
}

# Function to set optimum culling levels

optCull = function(ebv, weights, nSel){
  nInd = nrow(ebv)
  ebv = sweep(ebv[,1:2],2,weights,"*")
  t1 = order(ebv[,1],decreasing=TRUE)
  t2 = order(ebv[,2],decreasing=TRUE)
  val12 = val21 = numeric(nInd-nSel+1)
  #rait 1 followed by trait 2
  i=0
  for(n in nSel:nInd){
    i = i+1
    take = t1[1:n] #Selection on trait 1
    take = (t2[t2%in%take])[1:nSel] #Selection on trait 2
    val12[i] = mean(rowSums(ebv[take,1:2]))
  }
  best = max(val12)
  n = which.max(val12)+nSel-1
  take = t1[1:n] #Selection on trait 1
  take = (t2[t2%in%take])[1:nSel] #Selection on trait 2
  output = list(take=take, #Selected parents
                n=n, #Number selected in first round
                value=best) #Mean value of selection
  return(output)
}

##################################################################################
### Script example: scenario 1  ###
##################################################################################

#chosen economic weights
w = w1
#chosen accuracy
acc = 0.7
#number of replicates
replicates = 50

## Create objects to store records

SIGenMean <- list(NA)
SIGenVar <- list(NA)
SIGenicVar <- list(NA)
ICGenMean <- list(NA)
ICGenVar <- list(NA)
ICGenicVar <- list(NA)
ICRepRpop <- list(NA)
```

```r
SIRepRpop <- list(NA)
nStg2 <- list(NA)

## Run simulations

for(j in 1:replicates){
  cat("rep ",j,"\n")


  #### Creating Parents ####

  # Generate initial haplotypes
  founderpop = runMacs(nInd=50,
                       nChr=10,
                       segSites=nQtl,
                       inbred=TRUE,
                       species="WHEAT")

  SP = SimParam$new(founderpop)
  SP$addTraitAD(nQtlPerChr = nQtl, mean=initMeanG, var=initVarG, corA = corA,
                meanDD=0, corDD=corDD)
  addIndexTrait(weights = w)

  Parents = newPop(founderpop)
  Parents = setPheno(Parents,varE=varE,reps=1)

  #### Initialization and Burn-in ####

  #Set initial trials with unique individuals
  for(year in 1:3){

    if(year<=3){
      #Year 1
      F1 = randCross(Parents,nPop)

    }
    if(year<=2){
      #Year 2
      F1Pop = F1
      F1Pop = getEBV(pop = F1Pop, accuracy = acc, weights = w)

    }

    if(year<=1){
      #Year 3

      #finding the optimum number to be selected
      optimum = optCull(F1Pop@ebv, weights = w, nSel = nParents)

      #Stage2 = first culling
      Stage2 = selectInd(F1Pop, optimum$n, use = "ebv", trait = 1)

      #For selection on the index trait to be stalled and cycle time kept the same
```

```r
    #F1Pop moves to F1Pop2
    F1Pop2 = F1Pop

  }

}

## BURN-IN: Cycle years to make more advanced parents

#Create objects to track records
SIMean <- matrix(NA, 80, 3) #nrows= total number of years (burn-in + future breeding)
SIVar <- matrix(NA, 80, 3) #ncol = total number of traits (component traits + Index)
SIGenic <- matrix(NA, 80, 3)
ICMean <- matrix(NA, 80, 3)
ICVar <- matrix(NA, 80, 3)
ICGenic <- matrix(NA, 80, 3)
SIRpop <- list(NA)
ICRpop <- list(NA)
numStage2 <- data.frame(nSel = 1:80)

for(year in 1:20){ #Change to any number of desired years
  cat("Burn in: year ",year,"\n")
  ###Pick new inbred parents

  #Independent culling (IC): select from Stage 2.
  Parents = selectInd(Stage2,nParents, use = "ebv", trait = 2)

  ###Advances a year

  #Year 3

  #Find optimum
  optimum = optCull(F1Pop@ebv, weights = w, nSel = nParents)

  #Stage2: first culling
  Stage2 = selectInd(F1Pop, optimum$n, use = "ebv", trait = 1)

  #For selection on the index trait to be stalled and cycle time kept the same
  #F1Pop moves to F1Pop2
  F1Pop2 = F1Pop

  #Year 2
  F1Pop = F1
  F1Pop = getEBV(pop = F1Pop, accuracy = acc, weights = w)

  #Year 1
  F1 = randCross(Parents,nPop)

  #Record Results
  SIMean[year,] <- meanG(Parents)
  SIVar[year,] <- t(matrix(diag(varG(Parents))))
  SIGenic[year,] <- genicVarG(Parents)
  ICMean[year,] <- meanG(Parents)
```

```r
  ICVar[year,] <- t(matrix(diag(varG(Parents))))
  ICGenic[year,] <- genicVarG(Parents)

  SIRpop[[year]] <- data.frame(id = F1Pop@id, gv1 = F1Pop@gv[,1], gv2 = F1Pop@gv[,2])
  ICRpop[[year]] <- data.frame(id = F1Pop@id, gv1 = F1Pop@gv[,1], gv2 = F1Pop@gv[,2])

  numStage2[year,1] <- optimum$n

}

######### Breeding IC for 60 years ########

#Create pop objects for IC only
ICParents <- Parents
ICStage2 <- Stage2
ICF1Pop <- F1Pop
ICF1 <- F1

for(year in 1:60){ #Change to any number of desired years
  ###Pick new inbred parents
  cat("Future breeding: year ",year,"\n")

  #Independent culling (IC): select from Stage 2
  ICParents = selectInd(ICStage2,nParents, use = "ebv", trait = 2)

  ###Advances a year
  #Year 3
  optimum = optCull(ICF1Pop@ebv, weights = w, nSel = nParents)

  #Stage2: first culling
  ICStage2 = selectInd(ICF1Pop,optimum$n, use = "ebv", trait = 1)

  #Year 2
  ICF1Pop = ICF1
  ICF1Pop = getEBV(pop = ICF1Pop, accuracy = acc, weights = w)

  #Year 1
  ICF1 = randCross(ICParents,nPop)

  #Recording results

  ICMean[year+20,] <- meanG(ICParents)
  ICVar[year+20,] <- t(matrix(diag(varG(ICParents))))
  ICGenic[year+20,] <- genicVarG(ICParents)
  ICRpop[[year+20]] <- data.frame(id = ICF1Pop@id, gv1 = ICF1Pop@gv[,1],
                                  gv2 = ICF1Pop@gv[,2])
  numStage2[year+20,1] <- optimum$n

}

#IC Results for each replicate

ICGenMean[[j]] <- ICMean
```

```
  ICGenVar[[j]] <- ICVar
  ICGenicVar[[j]] <- ICGenic
  ICRepRpop[[j]] <- ICRpop
  nStg2[[j]] <- numStage2

  ######## Breeding SI for 60 years ########

  #Create pop objects for SI only
  SIF1Pop2 <- F1Pop2
  SIParents <- Parents
  SIStage2 <- Stage2
  SIF1Pop <- F1Pop
  SIF1 <- F1

  for(year in 1:60){ #Change to any number of desired years
    cat("Future breeding: year ",year,"\n")

    #Pick new inbred parents
    #Use @pheno, which has the values of the index (all columns/traits are the same)
    SIParents = selectInd(SIF1Pop2, nParents, use = "ebv", trait = 3)

    ###Advances a year
    #Year 3

    #For selection on the index trait to be stalled and cycle time kept the same
    #F1Pop moves to F1Pop2
    SIF1Pop2 = SIF1Pop

    #Year 2
    SIF1Pop = SIF1
    SIF1Pop = getEBV(pop = SIF1Pop, accuracy = acc, weights = w)

    #Year 1
    SIF1 = randCross(SIParents, nPop)

    #Recording results
    SIMean[year+20,] <- meanG(SIParents)
    SIVar[year+20,] <- t(matrix(diag(varG(SIParents))))
    SIGenic[year+20,] <- genicVarG(SIParents)
    SIRpop[[year+20]] <- data.frame(id = SIF1Pop@id, gv1 = SIF1Pop@gv[,1],
                                    gv2 = SIF1Pop@gv[,2])

  }

  #SI Results for each replicate

  SIGenMean[[j]] <- SIMean
  SIGenVar[[j]] <- SIVar
  SIGenicVar[[j]] <- SIGenic
  SIRepRpop[[j]] <- SIRpop

}
```
