# Supplementary material for: Long-term comparison between index selection and optimal independent culling in plant breeding programs with genomic prediction
Source: PLoS One. 2021 May 10;16(5):e0235554. doi: 10.1371/journal.pone.0235554 (PMC8109766; doi:10.1371/journal.pone.0235554)
Supplement: Attachment — Submitted filename: PONE-D-20-04603_reviewer.pdf [file pone.0235554.s003.pdf]

PLOS ONE

An economic selection index should be used instead of independent culling in plant breeding programs with genomic selection

--Manuscript Draft--

**Manuscript Number:**	PONE-D-20-04603
**Article Type:**	Research Article
**Full Title:**	An economic selection index should be used instead of independent culling in plant breeding programs with genomic selection
**Short Title:**	Comparison of multi trait selection methods for breeding programs deploying genomic selection
**Corresponding Author:**	Lorena G Batista<br>University of Edinburgh<br>Edinburgh, Edinburgh UNITED KINGDOM
**Keywords:**	
**Abstract:**	In the context of genomic selection, we evaluated and compared recurrent selection breeding programs using either index selection or independent culling for selection of parents. We simulated a clonally propagated crop breeding program for 20 cycles of selection using either independent culling or an economic selection index with two unfavourably correlated traits under selection. Cycle time from crossing to selection of parents was kept the same for both strategies. Our results demonstrate that accurate knowledge of the economic importance of traits is essential even when performing independent culling. This is because independent culling achieved its optimum genetic gain when the culling threshold for each trait varied accordingly to the economic importance of the traits. When gains from independent culling were maximised, the efficiency of converting genetic diversity into genetic gain of both selection methods were equivalent. When the same proportion selected of 10% for each trait was used instead of optimal culling levels, index selection was 10%, 128% and 310% more efficient than independent culling when the second trait had a relative economic importance of 1.0, 2.5 and 5.0, respectively. Given the complexity of estimating optimal culling levels and the fact that the gains achieved with independent culling are, at most, equivalent to index selection, the use of an economic selection index is recommended for multi-trait genomic selection.
**Order of Authors:**	Lorena G Batista
	Robert Chris Gaynor
	Gabriel R.A. Margarido
	Tim Byrne
	Peter Amer
	Gregor Gorjanc
	John M Hickey
**Additional Information:**	
**Question**	**Response**
**Financial Disclosure**<br><br>Enter a financial disclosure statement that describes the sources of funding for the work included in this submission. Review the submission guidelines for detailed requirements. View published research articles from *PLOS ONE* for specific	LGB was supported by the Coordenação de Aperfeiçoamento de Pessoal de Nível Superior (CAPES, Computational Biology Programme, Grant No. BEX 0043/17-6). JMH, RCG and GG acknowledge the financial support from BBSRC and KWS UK, RAGT Seeds Ltd., Elsoms Wheat Ltd and Limagrain UK for the project "GplusE: Genomic selection and Environment modelling for next generation wheat breeding" (grants BB/L022141/1 and BB/L020467/1).

Powered by Editorial Manager® and ProduXion Manager® from Aries Systems Corporation

examples.<br><br>This statement is required for submission and **will appear in the published article** if the submission is accepted. Please make sure it is accurate.<br><br>**Unfunded studies**<br>Enter: *The author(s) received no specific funding for this work.*<br><br>**Funded studies**<br>Enter a statement with the following details:<br>• Initials of the authors who received each award<br>• Grant numbers awarded to each author<br>• The full name of each funder<br>• URL of each funder website<br>• Did the sponsors or funders play any role in the study design, data collection and analysis, decision to publish, or preparation of the manuscript?<br>• **NO** - Include this sentence at the end of your statement: *The funders had no role in study design, data collection and analysis, decision to publish, or preparation of the manuscript.*<br>• **YES** - Specify the role(s) played.<br><br>* typeset	
**Competing Interests**<br><br>Use the instructions below to enter a competing interest statement for this submission. On behalf of all authors, disclose any competing interests that could be perceived to bias this work—acknowledging all financial support and any other relevant financial or non-financial competing interests.<br><br>This statement **will appear in the published article** if the submission is accepted. Please make sure it is accurate. View published research articles from *PLOS ONE* for specific examples.	The authors have declared that no competing interests exist.

**NO authors have competing interests**<br><br>Enter: *The authors have declared that no competing interests exist.*<br><br>**Authors with competing interests**<br><br>Enter competing interest details beginning with this statement:<br><br>*I have read the journal's policy and the authors of this manuscript have the following competing interests: [insert competing interests here]*<br><br>* typeset	
**Ethics Statement**<br><br>Enter an ethics statement for this submission. This statement is required if the study involved:<br><br>• Human participants<br>• Human specimens or tissue<br>• Vertebrate animals or cephalopods<br>• Vertebrate embryos or tissues<br>• Field research<br><br>Write "N/A" if the submission does not require an ethics statement.<br><br>General guidance is provided below. Consult the submission guidelines for detailed instructions. **Make sure that all information entered here is included in the Methods section of the manuscript.**	N/A

**Format for specific study types**<br><br>**Human Subject Research (involving human participants and/or tissue)**<br>• Give the name of the institutional review board or ethics committee that approved the study<br>• Include the approval number and/or a statement indicating approval of this research<br>• Indicate the form of consent obtained (written/oral) or the reason that consent was not obtained (e.g. the data were analyzed anonymously)<br><br>**Animal Research (involving vertebrate animals, embryos or tissues)**<br>• Provide the name of the Institutional Animal Care and Use Committee (IACUC) or other relevant ethics board that reviewed the study protocol, and indicate whether they approved this research or granted a formal waiver of ethical approval<br>• Include an approval number if one was obtained<br>• If the study involved *non-human primates*, add *additional details* about animal welfare and steps taken to ameliorate suffering<br>• If anesthesia, euthanasia, or any kind of animal sacrifice is part of the study, include briefly which substances and/or methods were applied<br><br>**Field Research**<br><br>Include the following details if this study involves the collection of plant, animal, or other materials from a natural setting:<br>• Field permit number<br>• Name of the institution or relevant body that granted permission	
**Data Availability**<br><br>Authors are required to make all data underlying the findings described fully available, without restriction, and from the time of publication. PLOS allows rare exceptions to address legal and ethical concerns. See the PLOS Data Policy and FAQ for detailed information.	Yes - all data are fully available without restriction

A Data Availability Statement describing where the data can be found is required at submission. Your answers to this question constitute the Data Availability Statement and **will be published in the article**, if accepted.<br><br>**Important:** Stating 'data available on request from the author' is not sufficient. If your data are only available upon request, select 'No' for the first question and explain your exceptional situation in the text box.<br><br>Do the authors confirm that all data underlying the findings described in their manuscript are fully available without restriction?	
**Describe where the data may be found in full sentences. If you are copying our sample text, replace any instances of XXX with the appropriate details.**<br><br>• If the data are **held or will be held in a public repository**, include URLs, accession numbers or DOIs. If this information will only be available after acceptance, indicate this by ticking the box below. For example: *All XXX files are available from the XXX database (accession number(s) XXX, XXX.*).<br>• If the data are all contained **within the manuscript and/or Supporting Information files**, enter the following: *All relevant data are within the manuscript and its Supporting Information files.*<br>• If neither of these applies but you are able to provide **details of access elsewhere**, with or without limitations, please do so. For example:<br><br>*Data cannot be shared publicly because of [XXX]. Data are available from the XXX Institutional Data Access / Ethics Committee (contact via XXX) for researchers who meet the criteria for access to confidential data.*<br><br>*The data underlying the results presented in the study are available from (include the name of the third party*	All relevant data are within the manuscript and its Supporting Information files.

and contact information or URL).<br>• This text is appropriate if the data are owned by a third party and authors do not have permission to share the data.<br><br>* typeset	
Additional data availability information:	

Click here to download Manuscript BatistaEtAl2020_plos.docx

An economic selection index should be used instead of independent culling in plant breeding programs with genomic selection

Lorena G. Batista*, R. Chris Gaynor, Gabriel R. A. Margarido, Tim Byrne, Peter Amer, Gregor Gorjanc, John M. Hickey

Lorena G. Batista and Gabriel R.A. Margarido: “Luiz de Queiroz” College of Agriculture (University of São Paulo), Piracicaba, SP, 13418-900, Brazil. R. Chris Gaynor, Gregor Gorjanc and John Hickey: The Roslin Institute and Royal (Dick) School of Veterinary Studies, University of Edinburgh, Easter Bush Campus Research Centre, Midlothian EH25 9RG, UK. Tim Byrne: Abacusbio, Roslin Innovation Centre, University of Edinburgh, Easter Bush Campus Research Centre, Midlothian EH25 9RG, UK. Peter Amer: Abacusbio, Public Trust Building, Moray Place, Dunedin 9016, New Zealand

Received ______________. * Corresponding author: loregbatista@gmail.com

Abstract

In the context of genomic selection, we evaluated and compared recurrent selection breeding programs using either index selection or independent culling for selection of parents. We simulated a clonally propagated crop breeding program for 20 cycles of selection using either independent culling or an economic selection index with two unfavourably correlated traits under selection. Cycle time from crossing to selection of parents was kept the same for both strategies. Our results demonstrate that accurate knowledge of the economic importance of traits is essential even when performing independent culling. This is because independent culling achieved its optimum genetic gain when the culling threshold for each trait varied accordingly to the economic importance of the traits. When gains from independent culling were maximised, the efficiency of converting genetic diversity into genetic gain of both selection methods were equivalent. When the same proportion selected of 10% for each trait was used instead of optimal culling levels, index selection was 10%, 128% and 310% more efficient than independent culling when the second trait had a relative economic importance of 1.0, 2.5 and 5.0, respectively. Given the complexity of estimating optimal culling levels and the fact that the gains achieved with independent culling are, at most, equivalent to index selection, the use of an economic selection index is recommended for multi-trait genomic selection.

Introduction

Crop breeding seeks to develop improved cultivars. Besides high yield levels, a successful cultivar in many crops must meet minimal standards for several other traits that are economically important, such as pest and disease resistance and product quality. Traits are often unfavourably correlated with each other (e.g., 1–5). When traits are antagonistically correlated, selection for one trait causes an undesired economic response in the other trait (6,7). This makes breeding to simultaneously improve multiple traits complicated.

Independent culling and the use of a selection index are two commonly used methods in plant breeding programs for selecting on multiple traits (7). Independent culling involves establishing minimum standards (i.e., culling levels) for each trait and only selecting individuals that meet these minimum standards. The thresholds can be set according to a specific selection intensity or a specific value, such as a value relative to an agronomic check. The application of independent culling can be on multiple traits simultaneously or on individual traits sequentially. The selection index method involves selection for all traits simultaneously based on a linear or non-linear combination of individual traits weighted by their importance for the breeding objective (8).

Theoretically, the selection index is the most effective method of selection for multiple traits (8–10). Independent culling is less effective than index selection because, when strictly applied, it will not select individuals below the threshold for only one trait despite being exceptional for all other traits, while the use of a selection index makes it possible to retain those individuals (7). However, independent culling can achieve nearly equivalent effectiveness using optimised thresholds (11).

When cost is considered, independent culling can be more efficient than a selection index (11). This is because independent culling does not require phenotypes for all individuals and traits at one time, whereas strict application of a selection index requires phenotypes for all traits. This benefit is particularly valuable to plant breeders, because early stages of the breeding program often have a very large number of individuals. Phenotyping all individuals for all traits is likely to be logistically and financially infeasible. For example, some traits have a high measurement cost, such as bread quality in wheat, so that they cannot be measured on a large number of individuals. Further, some traits can only be measured on older plants, such as lifetime production in sugarcane, or on a plot or group basis. Delaying selection until these traits become available would be effectively equivalent to random selection, because the breeder would have to reduce the overall size of the early stage. Thus, practical constraints require at least some use of independent culling on traits that can be phenotyped simply/quickly and at a lower cost in breeding programs utilising phenotypic selection.

The use of genomic selection in plant breeding may render the cost efficiency benefit of independent culling obsolete if all early generation individuals are genotyped. This is because genomic selection allows for accurate prediction of all traits at once (12). While genotyping all early generation individuals is not standard in most current breeding programs, it may become so in the future. This is likely to be the case if breeding programs adopt a two-part strategy to breeding that explicitly splits breeding programs into a rapid cycling, genomic selection guided, population improvement part tasked with developing new germplasm and a product development part focused on developing new varieties. Simulations of these breeding programs suggest they can deliver considerably more genetic gain than more conventional breeding programs (13).

Several studies have already discussed the benefits of incorporating genomic selection

strategies into crop breeding programs (13–16). In addition, other studies have demonstrated that combining index selection and genomic prediction can increase genetic gain in breeding programs (17,18) and, in the long term, even higher genetic gains can be obtained when multi-trait optimization strategies that also control for the loss of genetic diversity are used (19). However, differences in how multi-trait selection methods can affect not only genetic gain but other population parameters such as genetic diversity and genetic correlations over several cycles of recurrent selection have not yet been thoroughly investigated. In order to provide a more detailed account of population dynamics in the genomic selection framework, we used simulations of recurrent breeding programs to evaluate and compare both index selection and the independent culling method for 20 cycles of selection. The purpose of these simulations was to quantify the magnitude of the difference between optimally set independent culling levels and an optimal selection index. The simulations also investigated the sensitivity of independent culling to sub-optimal culling levels.

Material and Methods

Stochastic simulations of entire breeding programs for multiple traits were used to compare the genetic gains in a breeding program using independent culling levels and a breeding program using an economic selection index for selection of parents. In the independent culling approach, selection was performed for one trait at a time at each stage of selection. A clonally propagated crop species was considered. Generally, in breeding programs for clonally propagated species, several crosses are performed between highly heterozygous hybrids, and all the genotypes in the resulting F_1 progenies are candidate clones to be released as cultivars or used as parents in the next breeding cycle (20). The methods were compared using the average of fifty replicates, each replicate consisting of: i) a burn-in phase shared by both strategies so that each strategy had an

identical, realistic starting point; and ii) an evaluation phase that simulated future breeding with different breeding strategies. The burn-in phase consisted of 20 years of breeding using independent culling for the selection of parents and the evaluation phase consisted of 20 cycles of selection using either independent culling or index selection.

Genome sequence

For each replicate, a genome consisting of 10 chromosome pairs was simulated for the hypothetical clonally propagated plant species. These chromosomes were assigned a genetic length of 1.43 Morgans and a physical length of 8×10^8 base pairs. Sequences for each chromosome were generated using the Markovian Coalescent Simulator (21) and AlphaSimR (22). Recombination rate was inferred from genome size (i.e. 1.43 Morgans / 8×10^8 base pairs = 1.8×10^{-9} per base pair), and mutation rate was set to 2×10^{-9} per base pair. Effective population size was set to 50, with linear piecewise increases to 1,000 at 100 generations ago, 6,000 at 1,000 generations ago, 12,000 at 10,000 generations ago, and 32,000 at 100,000 generations ago.

Founder genotypes

Simulated genome sequences were used to produce 50 founder genotypes. These founder genotypes served as the initial parents in the burn-in phase. This was accomplished by randomly sampling gametes from the simulated genome to assign as sequences for the founders. Sites that were segregating in the founders' sequences were randomly selected to serve as 1,000 causal loci per chromosome (10,000 across the genome in total). To simulate genetic correlations between traits, the traits were treated as pleiotropic and the additive effects of the causal loci alleles were sampled from a multivariate normal distribution with mean $\mu = \begin{bmatrix} 0 \\ 0 \end{bmatrix}$ and desired values of correlation.

Estimated breeding values

The true genetic value of the simulated traits was determined by the summing of its causal loci allele effects. The matrix **E** with the estimated breeding values of the traits for each individual in the population was obtained according to the formula:

$$\mathbf{E} = \mathbf{Y}\mathbf{P}^{-1}\mathbf{G}$$

Where **Y** is the matrix of phenotypes simulated by adding random error to the true genetic values of the traits, where rows correspond to individuals in the population and columns correspond to traits. The random error was sampled from a multivariate normal distribution with mean $\mu = \begin{bmatrix} 0 \\ 0 \end{bmatrix}$ and zero covariance, with variance values tuned to achieve a target level of accuracy (r), which we define as the correlation between true and estimated breeding values. **P** is the phenotypic variance-covariance matrix of the traits, and **G** is the genetic variance-covariance matrix for the traits.

Breeding methods

The simulations modelled breeding for two component traits (T1 and T2) that were improved using either independent culling or an economic selection index. With both strategies, an F_1 population of 5,000 individuals was generated by randomly crossing the individuals in the crossing block (Parents). With independent culling, selection was carried out in two stages: a proportion of individuals was selected first based on T1 and then, from this proportion, the parents of the next breeding cycle were selected based on T2. With the selection index approach, the F_1 individuals with the highest values for the index trait were selected as parents of the next breeding cycle. The index trait was the sum of the estimated breeding values for each trait weighted by their economic importance. The number of selected parents (50 parents) and the cycle time from crossing to selection of new parents was kept the same for both strategies, so the comparisons

between them reflect only the differences due to the method of selection. The overall selection scheme used for each method of selection is shown in Supplementary material 1 (Fig S1.1). For simulation of breeding programs, we used the R package AlphaSimR (22).

Simulated scenarios

The selection index and independent culling methods were compared in a set of scenarios that aimed to assess the relative performance of the methods under different levels of accuracy of selection, and relative economic importance of T2. We were only interested in investigating the relative performance of selection methods under challenging conditions for multi trait selection. Hence, only an unfavourable genetic correlation between traits was simulated. A summary of all simulated scenarios we used in this study is shown in Table 1.

Table 1. Summary of parameters simulated in all comparison scenarios of recurrent selection breeding programs using either independent culling or selection index with two traits

Scenario	Selected Proportion		Relative economic importance of Trait 2	Accuracy
	Trait 1	Trait 2		
1	Optimum	Optimum	1.0	0.3
2	Optimum	Optimum	1.0	0.5
3	Optimum	Optimum	1.0	0.99
4	Optimum	Optimum	1.0	0.7
5	Optimum	Optimum	2.5	0.7
6	Optimum	Optimum	5.0	0.7
7	10%	10%	1.0	0.7
8	10%	10%	2.5	0.7
9	10%	10%	5.0	0.7

For one set of scenarios we simulated four levels of accuracy (0.3, 0.5, 0.7, and 0.99), assigned the same economic importance for both traits. In another set of scenarios, we varied the relative economic importance of T2, but fixed selection accuracy to 0.7. Here, three levels of relative economic importance were simulated. T1 was given an economic importance of 1.0 and T2 an economic importance of either 1.0, 2.5 or 5.0. For each level of relative economic importance, we simulated: i) scenarios where the proportion selected was the same (10%) when

selecting for both traits, and ii) scenarios where the proportions selected were set to achieve optimal culling levels (i.e., optimal independent culling). To achieve optimal culling levels, in each cycle of selection we chose the proportion selected for each trait that maximised the genetic gain. Thus, over the cycles of selection, when using optimal culling levels, instead of a fixed proportion selected of 10%, the proportion selected for each trait varied between cycles. To find the optimal proportions at each cycle, we fixed the number of parents selected (50 parents) and found the number of individuals to be selected in the first culling stage that maximized parents' economic value (i.e., index trait).

Comparison

The comparisons were made in terms of: i) genetic gain ii) genetic diversity, iii) the efficiency of converting genetic diversity into genetic gain for the index; and iv) genetic correlation between traits. For genetic gain and genetic diversity, we report values based on the individuals in the crossing block (parents) at each cycle of selection. We measured genetic gain as the increment in genetic mean (average of true genetic values) compared to the genetic mean in year 20. We measured genetic diversity with genetic standard deviation and genic standard deviation. We calculated genetic standard deviation as standard deviation of true genetic values. We calculated genic standard deviation as $\sigma_a = \sqrt{2\sum_{i=1}^{n_q} p_i(1-p_i)\alpha_i^2}$, where n_q is the number of causal loci and p_i and α_i are, respectively, allele frequency and allele substitution effect at the *i*-th causal locus.

To measure efficiency, genetic mean and genic standard deviation were standardized to mean zero and unit standard deviation in year 20. We measured efficiency of converting genetic diversity into genetic gain by regressing the achieved genetic mean ($y_t = \left(\mu_{a_t} - \mu_{a_{20}}\right)/\sigma_{a_{20}}^2$) on lost genetic diversity ($x_t = 1 - \sigma_{a_t}/\sigma_{a_{20}}$), i.e., $y_t = \alpha + bx_t + e_t$, where b is efficiency (23). We estimated efficiency with robust regression using function rlm() in R (24).

For genetic correlation, we report the correlation between the true genetic values of T1 and T2. We calculated this metric on the individuals in the F_1 population at each cycle of selection.

Results

Overall the results show that index selection provided consistent genetic gains and was equivalent to independent culling in terms of genetic gains and efficiency when optimal culling levels were used. Index selection performed better than independent culling in scenarios where independent culling levels were suboptimal.

We have structured the description of the results in two parts, corresponding to how the relative performance of the selection methods was affected by: i) the accuracy of selection, and ii) the relative economic importance of traits.

Accuracy of selection

The results show that increases in accuracy accentuated the differences in the genotypes being selected by either independent culling or index selection. This is shown in Fig. 1, where the genotypes selected as parents by each selection method are highlighted. Lower levels of accuracy led to a more diffuse cluster of selected genotypes and, with increasing selection accuracy, the cluster of selected genotypes approached what was expected for each method of selection (7).

Fig 1. Scatterplots of true genetic values for Trait 1 (T1) and Trait 2 (T2) of the genotypes in the F1 population (grey) and genotypes selected as parents (orange) in the third cycle of selection using either independent culling (a) or a selection index (b) with different levels of accuracy

Fig. 2 shows the change in the genetic correlation between the component traits for both independent culling and index selection over 20 cycles of selection at different levels of accuracy. Both selection methods resulted in the correlation between traits becoming increasingly unfavourable over the cycles of selection. For both methods, the change in the genetic correlation was higher with higher values of accuracy. Compared to independent culling, index selection led

to larger changes in the genetic correlation between the two traits. After 20 cycles of selection with accuracy of 0.3, independent culling led to a genetic correlation that was 9% more unfavourable compared to the genetic correlation in cycle 0, while index selection led to a genetic correlation that was 17% more unfavourable compared to the genetic correlation in cycle 0. After 20 cycles of selection with accuracy of 0.99, independent culling led to a genetic correlation that was 29% more unfavourable compared to the genetic correlation in cycle 0, while index selection led to a genetic correlation that was 64% more unfavourable compared to the genetic correlation in cycle 0.

Fig 2. Change in genetic correlation (mean and 95% confidence interval) between traits in the F1 population over 20 cycles of selection using either optimal independent culling (IC) or a selection index (SI) with different levels of accuracy, and Trait 2 relative economic importance of 1.0

The change of genetic mean in parents for the component traits and the index trait over the cycles of selection using each method is shown in Fig. 3. For both methods, the genetic gains for the component traits and the index trait increased with higher values of accuracy. In general, the selection index method and independent culling with optimal culling levels led to equivalent genetic gains for the component traits and the index trait. Only in the scenario with 0.99 accuracy did index selection lead to a slightly higher genetic gain compared to that achieved with optimal independent culling. For the index trait, after 20 cycles of selection with accuracy of 0.99, index selection had a genetic gain 4% higher than the genetic gain achieved with independent culling.

Fig 3. Change in genetic mean for Trait 1 (T1), Trait 2 (T2) and Index Trait (Index) over 20 cycles of selection using either optimal independent culling (IC) or a selection index (SI) with different levels of accuracy, unfavourably correlated traits, and T2 relative economic importance of 1.0

Table 2 shows the genetic standard deviation of parents in cycle 20 and the loss in genetic standard deviation in cycle 20 compared to the genetic standard deviation in cycle 0 for the component traits and the index trait. The change of genetic diversity in parents for the component

traits and the index trait over the cycles of selection using each method is shown in Supplementary material 1 (Fig S1.2). For the component traits, when using index selection, the genetic standard deviation showed an initial increase in the first few cycles of selection followed by a gradual decrease in the subsequent cycles. When using independent culling, the decrease in the genetic standard deviation of the component traits was continual over the cycles of selection. Both of these trends were more obvious with increasing values of accuracy. For all values of accuracy, independent culling led to a higher loss in the genetic standard deviation of the component traits compared to the index selection. For T1 and T2, independent culling with accuracy of 0.3 led to a loss of genetic standard deviation that was 6% and 5% higher than the loss of genetic standard deviation observed for index selection, respectively. With accuracy of 0.99, for T1 and T2 independent culling led to a loss of genetic standard deviation that was 65% and 51% higher than the loss of genetic standard deviation observed for index selection, respectively. For the index trait, both methods led to equivalent values of genetic standard deviation. With accuracies of 0.3 and 0.99, index selection led to a loss in the genetic standard deviation of the index trait that was 3% higher compared to the loss of genetic standard deviation observed using independent culling, respectively.

Table 2. Mean genetic standard deviation (Genetic SD) of parents in cycle 20 and loss in genetic standard deviation in cycle 20 in comparison to the genetic standard deviation in cycle 0 (Loss over cycle 0) for trait 1 (T1), trait 2 (T2) and the index trait using either optimal independent culling or index selection with different levels of accuracy, unfavourably correlated traits, and T2 relative economic importance of 1.0

	**Independent culling**					
	**T1**		**T2**		**Index trait**	
**Accuracy**	**Genetic SD (cycle 20)**	**Loss over cycle 0**	**Genetic SD (cycle 20)**	**Loss over cycle 0**	**Genetic SD (cycle 20)**	**Loss over cycle 0**
0.3	3.51 (0.08)*	-17%	3.68 (0.08)	-16%	3.57 (0.06)	-22%
0.5	2.56 (0.06)	-30%	2.45 (0.04)	-28%	2.69 (0.05)	-32%
0.7	1.65 (0.04)	-42%	1.64 (0.03)	-37%	1.88 (0.04)	-45%
0.99	0.45 (0.01)	-68%	0.45 (0.01)	-55%	0.74 (0.02)	-62%
	**Index Selection**					
	**T1**		**T2**		**Index trait**	
**Accuracy**	**Genetic SD (cycle 20)**	**Loss over cycle 0**	**Genetic SD (cycle 20)**	**Loss over cycle 0**	**Genetic SD (cycle 20)**	**Loss over cycle 0**
0.3	3.80 (0.09)	-11%	4.00 (0.09)	-11%	3.66 (0.08)	-19%
0.5	3.19 (0.08)	-17%	3.19 (0.07)	-14%	2.57 (0.06)	-33%
0.7	2.69 (0.06)	-16%	2.60 (0.06)	-18%	1.86 (0.04)	-41%
0.99	1.93 (0.4)	-3%	1.91 (0.04)	-4%	0.51 (0.01)	-59%

* standard errors of the estimates are presented in parenthesis

Table 3 shows the genic standard deviation of parents in cycle 20 and the loss in genic standard deviation in cycle 20 compared to the genic standard deviation in cycle 0 for the component traits and the index trait. The values of genic standard deviation of T1, T2, and the index trait were equivalent. The highest difference between methods in the loss in genic standard deviation was 1% for all values of accuracy, except with accuracy of 0.99. With 0.99 accuracy, for T1, T2 and the index trait, index selection led to a loss in the genic standard deviation that was 3% higher compared to the loss of genic standard deviation observed using independent culling.

Table 3. Genic standard deviation (Genic SD) of parents in cycle 20 and loss in genic standard deviation in cycle 20 in comparison to the genic standard deviation in cycle 0 (Loss over cycle 0) for trait 1 (T1), trait 2 (T2) and the index trait using either optimal independent culling or index selection with different levels of accuracy, unfavourably correlated traits, and T2 relative economic importance of 1.0

	**Independent culling**					
	**T1**		**T2**		**Index trait**	
**Accuracy**	**Genic SD (cycle 20)**	**Loss over cycle 0**	**Genic SD (cycle 20)**	**Loss over cycle 0**	**Genic SD (cycle 20)**	**Loss over cycle 0**
0.3	3.94 (0.06)*	-15%	4.11 (0.07)	-15%	4.04 (0.05)	-16%
0.5	3.48 (0.06)	-24%	3.41 (0.05)	-24%	3.44 (0.04)	-25%
0.7	2.94 (0.04)	-34%	2.89 (0.04)	-34%	2.89 (0.04)	-34%
0.99	2.35 (0.04)	-42%	2.35 (0.04)	-42%	2.33 (0.04)	-43%
	**Index Selection**					
	**T1**		**T2**		**Index trait**	
**Accuracy**	**Genic SD (cycle 20)**	**Loss over cycle 0**	**Genic SD (cycle 20)**	**Loss over cycle 0**	**Genic SD (cycle 20)**	**Loss over cycle 0**
0.3	3.92 (0.06)	-16%	4.08 (0.07)	-16%	4.02 (0.05)	-16%
0.5	3.44 (0.06)	-25%	3.37 (0.05)	-25%	3.39 (0.05)	-26%
0.7	2.92 (0.05)	-34%	2.88 (0.05)	-34%	2.87 (0.04)	-35%
0.99	2.21 (0.04)	-45%	2.22 (0.04)	-45%	2.17 (0.03)	-46%

* standard errors of the estimates are presented in parenthesis

Relative economic importance of traits

Fig. 4 shows the efficiency of converting genetic diversity into genetic gain for the index trait when the relative economic importance of T2 varies. Independent culling was compared to index selection using either optimal culling levels or selection with the same proportion of plants selected (10%) for each trait. Index selection had the highest efficiency and most gain for all levels of economic importance. The efficiency and gain for optimal independent culling levels was nearly equivalent to index selection. The efficiency and gain for selecting the same proportion of plants for both traits was worse than index selection for all levels of relative economic importance. Index selection was 10%, 128% and 310% more efficient than independent culling using the same proportion of selected plants for relative economic importance of 1.0, 2.5 and 5.0, respectively.

Fig 4. Change of genetic mean and genic standard deviation for the index trait across 20 cycles of selection using either independent culling (IC) or a selection index (SI) under three levels of relative economic importance (REI) and using either the same proportion selected (10%) for Trait 1 (T1) and Trait 2 (T2) or optimal culling levels for each level of relative

economic importance of T2 (a); and proportion selected (mean and 95% confidence interval) for T1 used to achieve optimal culling levels over the 20 cycles of selection (b). Traits are unfavourably correlated (-0.5). Individual replicates are shown by thin lines and a mean regression with a time-trend arrow. Values of genetic mean and genic standard deviation shown are standardized to mean zero and unit standard deviation in cycle 0

Fig. 4 also shows the proportion of plant selected for T1 under optimal independent culling over the different levels of economic importance for T2. The mean proportion selected for T1 only varied slightly over the cycles of selection. The means were 29%, 93%, and 99% for relative economic importance of 1.0, 2.5, and 5.0, respectively. The variation about those means was largest with relative economic importance of 1.0 and smallest with relative economic importance of 5.0.

Discussion

This study evaluated and compared recurrent selection breeding programs that either use index selection or independent culling for the selection of parents by genomic selection. Overall the results show that using index selection is either better or equivalent to independent culling in this context. Index selection outperformed independent culling when sub-optimal culling levels were used. Our results demonstrate that accurately assessing the economic importance of the traits is essential regardless of the method of selection being used.

The main difference between index selection and independent culling is that, when using index selection, genotypes that are exceptional for one of the traits under selection are more likely to be selected even though their performance for other traits is average. This can be seen in Fig. 1, with the cluster of individuals selected as parents with the index method including individuals that are more contrasting for the two traits under selection compared to the individuals selected with independent culling. The main implications of this are in the way each method affects the correlation between traits and the genetic diversity over cycles of recurrent selection. We discuss

each of these aspects in the following two sections. In the third section, we discuss how the relative economic importance of the traits can affect the relative performance of the methods. Lastly, we discuss the implications of our results for modern plant breeding programs which deploy genomic selection.

Methods of selection and genetic correlation between traits

The results show that, after only a few cycles of selection, index selection generates F_1 populations with a more unfavourable genetic correlation between traits than the F_1 populations generated by independent culling (Fig 2). An explanation for the faster decrease of the genetic correlation observed with index selection is that the index is a linear combination of component traits. As shown by Bulmer (25), selection on a linear combination leads to negative covariances between components (i.e., Bulmer effect). Consequently, the same principle applies to the component traits and index selection, with index selection leading to an unfavourable genetic correlation between the component traits (26,27).

In general, genetic gains in multi-trait selection, regardless of the method of selection, are expected to be higher when the correlation between traits is favourable and lower when this correlation is unfavourable (9). As index selection generated F_1 populations with more unfavourable genetic correlation between traits than independent culling, the genetic gains for index selection were potentially lower than for independent culling. Nevertheless, despite index selection being carried out under increasingly unfavourable genetic correlations over the cycles, the genetic gains obtained for the index trait were equivalent to the gains obtained using independent culling (Fig. 3).

Unfavourable genetic correlations are the most challenging scenario for breeders. When

traits are unfavourably correlated, selection on one trait results in response in an undesired direction for the other trait. When these correlations are due to pleiotropy, they cannot be broken with repeated cycles of recombination. This case is likely pervasive in several crops, e.g., grain yield and protein content in cereal crops (28–30), quality and disease resistance in forage crops (31), and yield and disease resistance in barley (32). However, the extent of genetic correlation and pleiotropy in these examples is unknown because unfavourable genetic correlations between the traits could also be, at least partly, induced by selection, as demonstrated in this study.

Methods of selection and genetic diversity over cycles of selection

According to Bulmer (25), reduction in the genetic variance due to selection stems mostly from the build-up of negative linkage disequilibrium between causal loci when selection is performed. This can be seen by comparing genetic and genic variation (Table 2 and Table 3, respectively). Genic variation is a function of the allele frequencies and the allele substitution effect only, and thus is not affected by changes in linkage disequilibrium. The results in Table 3 show that the loss of genic standard deviation of the component traits and index trait are not greatly affected by the method of selection. Also, the method of selection did not greatly affect the trait means, as shown in Fig. 3. This indicates that, in terms of allele frequencies, there was little difference in the parents selected by either independent culling or the selection index method in situations similar to our simulation. Therefore, the difference between the selection methods derives from how they induce and exploit linkage disequilibrium between the causal variants of the component traits. Specifically, as shown in Table 2, independent culling induced a greater degree of negative linkage disequilibrium between the causal variants of the component traits resulting in those traits having less genetic variation. A deviation from this result is expected with more intense selection schemes and more component traits selected in successive stages, which

would induce larger changes in allele frequencies due to drift. As a consequence, differences between index selection and independent culling would be accentuated. In a previous study (33), the authors simulated and compared wheat breeding programs using different selection strategies under high and low selection intensities. They observed index selection resulted in higher population coancestry over cycles of selection compared to independent culling, and the difference between methods increased in scenarios with high selection intensity. Their results indicate index selection leads to a higher loss of genic standard deviation.

Somewhat surprisingly, it is possible to make an argument for the superiority of independent culling relative to a selection index on the basis of the differences observed in linkage disequilibrium. This is because independent culling produced populations with nearly equivalent mean performance, but with more consistent performance between individuals, which is demonstrated by the lower variation observed for the component traits. This property could be beneficial from a management perspective if differences in the component traits require variations in management of individuals. Breeding for plant-architecture traits in outbreeding cultivars is a good example where this property might be valuable, as having more uniform plants in the field favours mechanical harvest. However, we believe this property is more of an academic curiosity than something that will have practical application.

For simplicity and ease of implementation, our simulations consider the same genetic architecture for both traits, with both traits being controlled by a high number (10,000) of causal loci with small additive effects. Under different circumstances, such as at least one of the traits being controlled by few causal loci with higher allele substitution effects, different results could be expected. The results for the two-locus model in (34) show that independent culling tends to eliminate genotypes that are homozygous for alleles with low effect for one of the traits. For one

pleiotropic causal locus, when both alleles are favourable for one trait and unfavourable for the other trait, both homozygous genotypes tend to be culled, and independent culling would select the heterozygous genotypes. If heterozygous genotypes were preferred, the fixation of alleles would be slower and, therefore, the loss in genic standard deviation would be lower. Our results indicate that, for highly polygenic traits, differences between methods of selection in the loss of genetic diversity are mostly due to changes in linkage disequilibrium as opposed to distinctive changes in allele frequencies. Therefore, in terms of conserving genetic diversity there was no obvious advantage for either method. Other strategies such as optimal-cross selection (23,33,35,36) or the multi-objective optimized approach (19) should be considered in order to optimize gains while also controlling the loss of genetic diversity over cycles of selection.

Economic importance of the traits

In general, when using the same selection intensity for both traits, the greater the difference in the economic importance of the traits, the better index selection will perform compared to independent culling (Fig. 4). This happens because there is a combination of selection intensities for each trait that maximizes the genetic gain when performing independent culling (8). Finding these selection intensities when selecting for two traits in two stages of selection is complex (37–40), and becomes even more complex with increasing number of traits and stages of selection (11,41,42).

The results in Fig. 4 show that independent culling approaches its maximal gain when a higher selection intensity is used for the trait with higher economic importance and a lower selection intensity is used for the trait with lower economic importance. In fact, when one trait had 5 times the economic importance of the other trait, the optimum was achieved when almost no selection was carried out for the less important trait. These results demonstrate that accurately

assessing the economic importance of the traits is essential even when independent culling is performed.

Regardless of the gains achieved with independent culling being maximised, when parents are selected based on an index, equivalent gains are achieved by simply summing the values of the traits weighted by their economic importance. Once the true economic weights of the traits are quantified, index selection is much simpler than independent culling when using these weights for optimizing the genetic gains in a plant breeding program.

Index selection in modern plant breeding programs that use genomic selection

There is little to no evidence suggesting plant breeders use analytical techniques to determine optimal independent culling thresholds and/or constructing selection indices in most plant breeding programs. More likely, the majority of breeders rely on their intuition for setting thresholds and constructing indices. Their decisions are likely guided by the performance of agronomic checks and are prone to fluctuations between seasons and individual breeders. This model has clearly been successful, because plant breeding programs have continued to deliver genetic gain. However, it is likely sub-optimal, and a more analytical approach should be adopted in the future.

The value of a more analytical approach becomes greater as genomic selection is more widely used. The results presented in this paper show a selection index is superior to independent culling when using genomic selection. These results are further supported by earlier theoretical work (8,9,43) and recent implementation of index selection in modern breeding programs of wheat (18) and Miscanthus (17). This indicates a clear preference for implementing selection indices in plant breeding.

The focus of plant breeders should be determining the economic weights for a selection index. In this paper the economic model used to select weights was implicitly assumed to be known and linear. The reality is that true economic model may be unknown to breeders and it is likely non-linear. The presence of a non-linear model does not pose a problem, because linear economic weights can be derived for improving the economic value of germplasm (44). However, this still requires defining the economic model, which constitutes the main challenge for using selection indices successfully.

The best way of determining economic weights has been a topic of extensive research and scientific debate (45–51). Recently, multi trait strategies have been proposed that suggest selection weights should be learned directly from the breeding population under selection. The authors in (52) proposed an eigen selection index method that do not require economic weights to be previously known and, instead, estimates them using genomic information from the breeding population. On the strategy proposed in (19), breeding choices are carried based on optimization of trade-offs across multiple traits as well as possible losses of genetic diversity in the population.

Given all the available tools, it is our opinion that plant breeders would benefit greatly from an increased emphasis on understanding and quantifying the economics of their species and using more analytical approaches when selecting for multiple traits. This would greatly aid breeders in getting the most out of genomic selection.

Conclusions

We evaluated and compared recurrent selection breeding programs using either independent culling or index selection for parent selection. The results show that, despite selection being carried out under unfavourable genetic correlations when using the selection index instead of independent culling, equivalent or higher genetic gains were achieved with index selection in

all simulated scenarios. In terms of genetic diversity, the differences between methods in the studied system were driven mostly by differences in the generation of linkage disequilibrium between causal loci induced and not differences in allele frequencies. When linkage disequilibrium was not considered, both methods were equivalent in terms of loss of genetic diversity, and the differences between methods in terms of efficiency of converting genetic diversity into genetic gains mostly reflected the differences in the genetic gains obtained with each method. To obtain higher genetic gains, accurately assessing the economic importance of the traits is essential even when independent culling is performed, as optimal culling levels should be determined in order for maximum gain to be achieved. Given that optimal culling levels are complex to estimate, once the economic importance of each trait is known, maximum genetic gains are more easily achieved with index selection. Therefore, the best choice for plant breeding programs is to select parents using an economic selection index.

Acknowledgements

This study was supported by the Coordenação de Aperfeiçoamento de Pessoal de Nível Superior (CAPES, Computational Biology Programme, Grant No. BEX 0043/17-6). The Roslin authors acknowledge the financial support from BBSRC and KWS UK, RAGT Seeds Ltd., Elsoms Wheat Ltd and Limagrain UK for the project "GplusE: Genomic selection and Environment modelling for next generation wheat breeding" (grants BB/L022141/1 and BB/L020467/1).

Conflict of interest

On behalf of all authors, the corresponding author states that there is no conflict of interest.

References

1. Kwon SH, Torrie JH. Heritability of and interrekationships among traits of two soybean population. 1964.

2. Meredith WR, Bridge RR. Breakup of Linkage Blocks in Cotton, Gossypium hirsutum L.1. Crop Science. 1971;11:695–8.

3. Erskine W, Williams PC, Nakkoul H. Genetic and environmental variation in seed yield, seed size and cooking quality of lentil. Field Crops Research. 1985;12:153–61.

4. Kato T, Takeda K. Associations among Characters Related to Yield Sink Capacity in Space-Planted Rice. Crop Science. 1996;36:1135–9.

5. Triboi E, Martre P, Girousse C, Ravel C, Triboi-Blondel AM. Unravelling environmental and genetic relationships between grain yield and nitrogen concentration for wheat. European Journal of Agronomy. 2006;25(2):108–18.

6. Falconer DS, Mackay TF, Frankham R. Introduction to Quantitative Genetics (4th edn). Trends in Genetics. 1996;12(7):280.

7. Bernardo R. Breeding for quantitative traits in plants. 1st ed. Woodbury: Stemma Press; 2010. 390 p.

8. Hazel LN, Lush JL. The Efficiency of Three Methods of Selection. Journal of Heredity. 1942;33(11):393–9.

9. Young S. A further examination of the relative efficiency of three methods of selection for genetic gains under less-restricted conditions. Genetical Research Cambridge. 1961;2(1961):106–21.

10. Pesek J, Baker RJ. Comparison of tandem and index selection in the modified pedigree method of breeding self-pollinated species. Canadian Journal of Plant Science. 1969 Nov;49(6):773–81.

11. Xu S, Muir WM. Multistage Selection for Genetic Gain by Orthogonal Transformation. Genetics. 1991 Nov;129(3):963–74.

12. Meuwissen THE, Hayes BJ, Goddard ME. Prediction of total genetic value using genome-wide dense marker maps. Genetics. 2001;157(4):1819–29.

13. Gaynor RC, Gorjanc G, Bentley AR, Ober ES, Howell P, Jackson R, et al. A Two-Part Strategy for Using Genomic Selection to Develop Inbred Lines. Crop Science. 2017;57:2372–86.

14. Bernardo R, Yu J (2007) Prospects for Genomewide Selection for Quantitative Traits in Maize. Crop Science 47:1082–1090. doi: 10.2135/cropsci2006.11.0690

15. Heffner EL, Sorrells ME, Jannink J-L (2009) Genomic Selection for Crop Improvement. Crop Science 49:1–12. doi: 10.2135/cropsci2008.08.0512

16. Hickey JM, Chiurugwi T, Mackay I, Powell W, Participants IGS in CBPW. Genomic prediction unifies animal and plant breeding programs to form platforms for biological discovery. Nature Genetics. 2017 Aug 30;49:1297.

17. Slavov GT, Davey CL, Bosch M, Robson PRH, Donnison IS, Mackay IJ. Genomic index selection provides a pragmatic framework for setting and refining multi-objective breeding targets in Miscanthus. Annals of Botany [Internet]. 2018 Oct 23 [cited 2019 Jun 9]; Available from: https://academic.oup.com/aob/advance-article/doi/10.1093/aob/mcy187/5142550

18. Michel S, Löschenberger F, Ametz C, Pachler B, Sparry E, Bürstmayr H. Simultaneous selection for grain yield and protein content in genomics-assisted wheat breeding. Theoretical and Applied Genetics. 2019 Jun;132(6):1745–60.

19. Akdemir D, Beavis W, Fritsche-Neto R, Singh AK, Isidro-Sánchez J. Multi-objective optimized genomic breeding strategies for sustainable food improvement. Heredity. 2019 May;122(5):672–83.

20. Grüneberg W, Mwanga R, Andrade M, Espinoza J. Selection methods. Part 5: Breeding clonally propagated crops. Plant Breeding and Farmer Participation. 2009;(April):275–322.

21. Chen GK, Marjoram P, Wall JD. Fast and flexible simulation of DNA sequence data. Genome research. 2009;19(1):136–142.

22. Gaynor RC, Gorjanc G, Wilson DL. AlphaSimR: An R Package for Breeding Program Simulations. Manuscr Prep.

23. Gorjanc G, Gaynor RC, Hickey JM. Optimal cross selection for long-term genetic gain in two- part programs with rapid recurrent genomic selection. bioRxiv. 2017;

24. Venables WN, Ripley BD. Modern Applied Statistics with S. Fourth Edi. New York: Springer; 2002.

25. Bulmer MG (1971) The effect of selection on genetic variability. 105:1–88

26. Tallis GM. Ancestral covariance and the Bulmer effect. Theoretical and Applied Genetics. 1987;73(6):815–20.

27. Itoh Y. Changes in genetic correlations by index selection. Genetics selection evolution GSE. 1991;23(4):301–8.

28. Duvick DN, Cassman KG. Post–Green Revolution Trends in Yield Potential of Temperate Maize in the North-Central United States. Crop Science. 1999;39:1622–30.

29. Rharrabti Y, Elhani S, Martos-Núñez V, García del Moral LF. Protein and Lysine Content, Grain Yield, and Other Technological Traits in Durum Wheat under Mediterranean Conditions. Journal of Agricultural and Food Chemistry. 2001 Aug 1;49(8):3802–7.

30. Rotundo JL, Borrás L, Westgate ME, Orf JH. Relationship between assimilate supply per seed during seed filling and soybean seed composition. Field Crops Research. 2009;112(1):90–6.

31. Casler MD, Vogel KP. Accomplishments and Impact from Breeding for Increased Forage Nutritional Value. Crop Science. 1999;39:12–20.

32. Smedegaard-Petersen V, Tolstrup K. The Limiting Effect of Disease Resistance on Yield. Annual Review of Phytopathology. 1985;23(1):475–90.

33. Cowling W, Li L. Turning the heat up on independent culling in crop breeding. 2018. 119–134 p.

34. Bennett G, Swiger L. Genetic variance and correlation after selection for two traits by index, independent culling levels and extreme selection. Genetics. 1980;94(3):763–75.

35. Clark SA, Kinghorn BP, Hickey JM, Van Der Werf JHJ. The effect of genomic information on optimal contribution selection in livestock breeding programs. Genetics Selection Evolution. 2013;45(1):1–8.

36. Woolliams JA, Berg P, Dagnachew BS, Meuwissen THE. Genetic contributions and their optimization. Journal of Animal Breeding and Genetics. 2015 Apr;132(2):89–99.

37. Young SSY, Weiler H. Selection for two correlated traits by independent culling levels. Journal of Genetics. 1960;57(2):329–38.

38. Namkoong G. Optimum Allocation of Selection Intensity in Two Stages of Truncation Selection. Biometrics. 1970 Sep;26(3):465.

39. Cotterill PP, James JW. Optimising two-stage independent culling selection in tree and animal breeding. Theoretical and Applied Genetics [Internet]. 1981 [cited 2018 Oct 19];59(2). Available from: http://link.springer.com/10.1007/BF00285891

40. Smith SP, Quaas RL. Optimal Truncation Points for Independent Culling-Level Selection Involving Two Traits. Biometrics. 1982 Dec;38(4):975.

41. Saxton AM. INDCULL Version 3.0: Independent Culling for Two or More Traits. Journal of Heredity. 1989 Mar 1;80(2):166–7.

42. Ducrocq V, Colleau JJ. Optimum truncation points for independent culling level selection on a multivariate normal distribution, with an application to dairy cattle selection. Genetics Selection Evolution. 1989 Jun 15;21(2):185.

43. Smith HF. A Discriminant Function for Plant Selection. Annals of Eugenics. 1936 Nov 1;7(3):240–50.

44. Goddard ME. Selection indices for non-linear profit functions. Theoret Appl Genetics. 1983 Mar 1;64(4):339–44.

45. Moav R. Economic evaluation of genetic differences. In: Agricultural genetics: selected topics. New York: Wiley; 1973. p. 319–52.

46. Brascamp EW, Smith C, Guy DR. Derivation of economic weights from profit equations. Animal Production. 1985;40:175–80.

47. Smith C, James JW, Brascamp EW. On the derivation of economic weights in livestock improvement. Animal Science. 1986;43(3):545–51.

48. Johnson BE, Dauer JP, Gardner CO. A model for determining weights of traits in simultaneous multitrait selection. Applied Mathematical Modelling. 1988 Dec;12(6):556–64.

49. Amer PR, Fox GC. Estimation of economic weights in genetic improvement using neoclassical production theory: an alternative to rescaling. Animal Science. 1992 Jun;54(3):341–50.

50. Liu Y-H, Dauer JP. A bicriteria linear programming model for determining linear utility functions in simultaneous multiple trait selection and classification. Applied Mathematical Modelling. 1996 Aug;20(8):572–8.

51. Aubry CA, Adams WT, Fahey TD. Determination of relative economic weights for multitrait selection in coastal Douglas-fir. 1998;28:7.

52. Ceron Rojas JJ, Crossa J. Linear selection indices in modern plant breeding. Springer; 2018.

Figure 1 Click here to download Figure Fig1.tiff

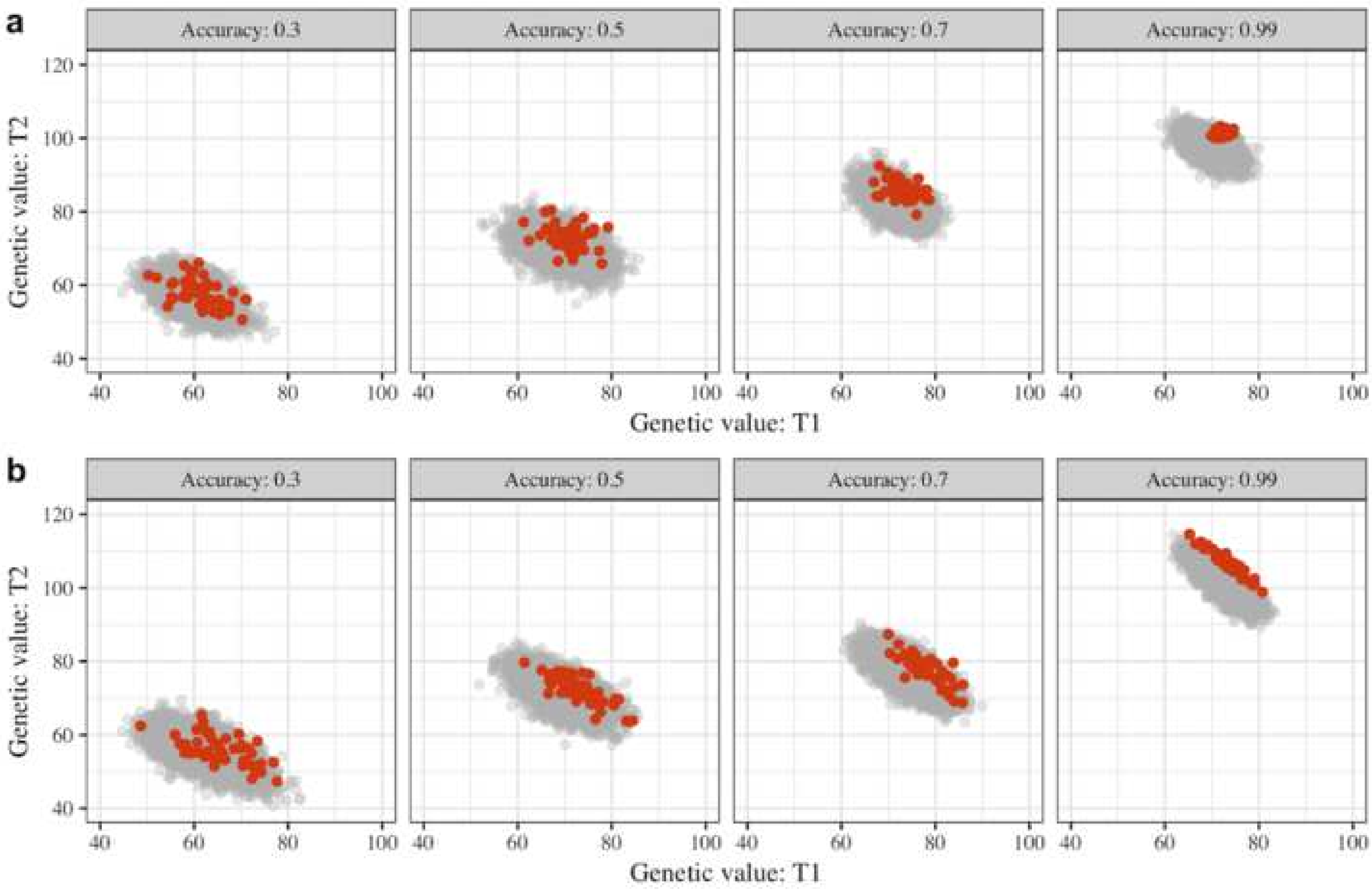

Figure 2

Click here to download Figure Fig2.tiff

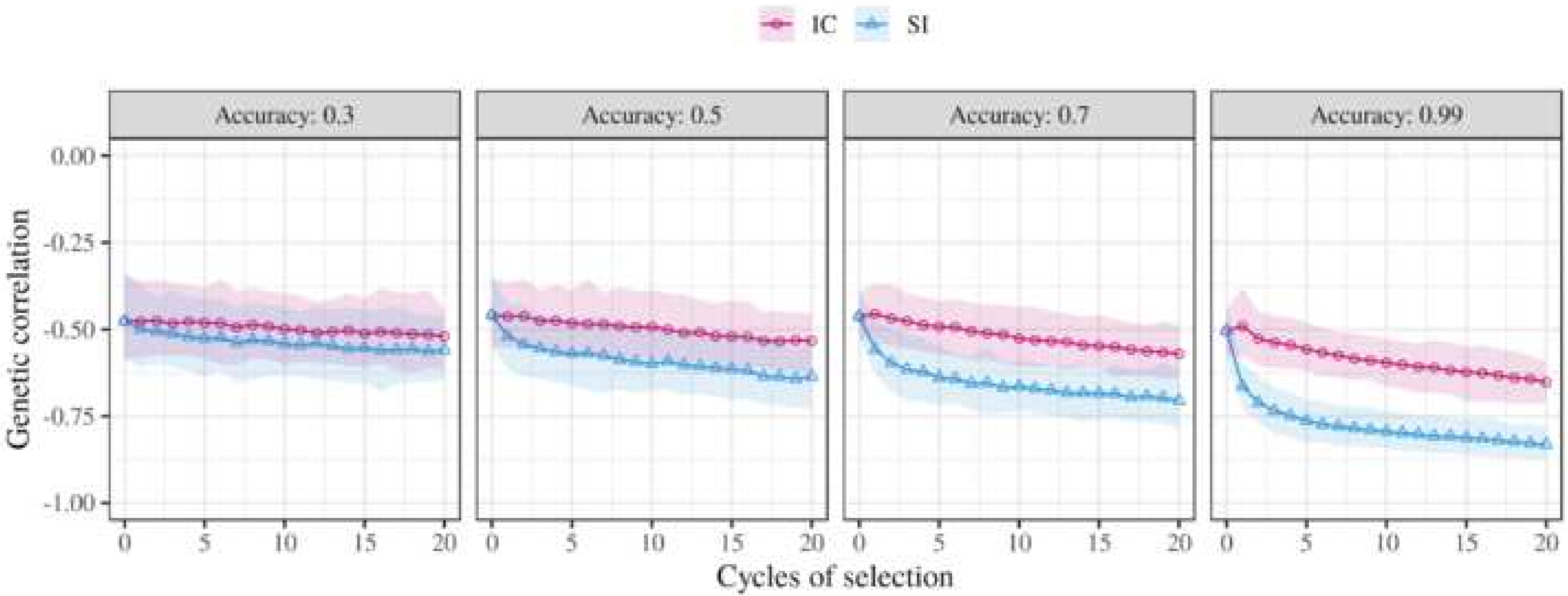

Figure 3

Click here to download Figure Fig3.tiff

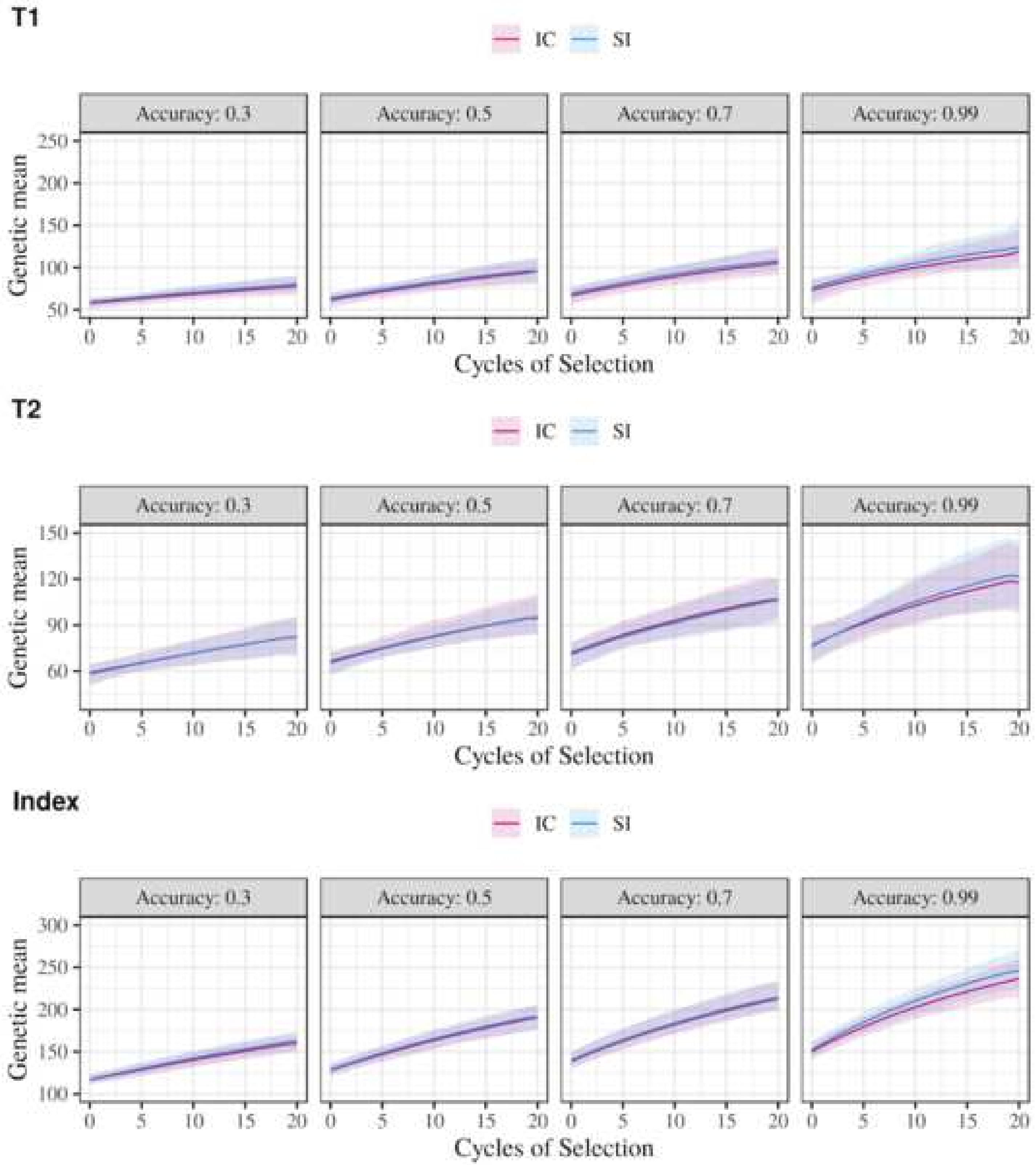

Figure 4

Click here to download Figure Fig4.tiff

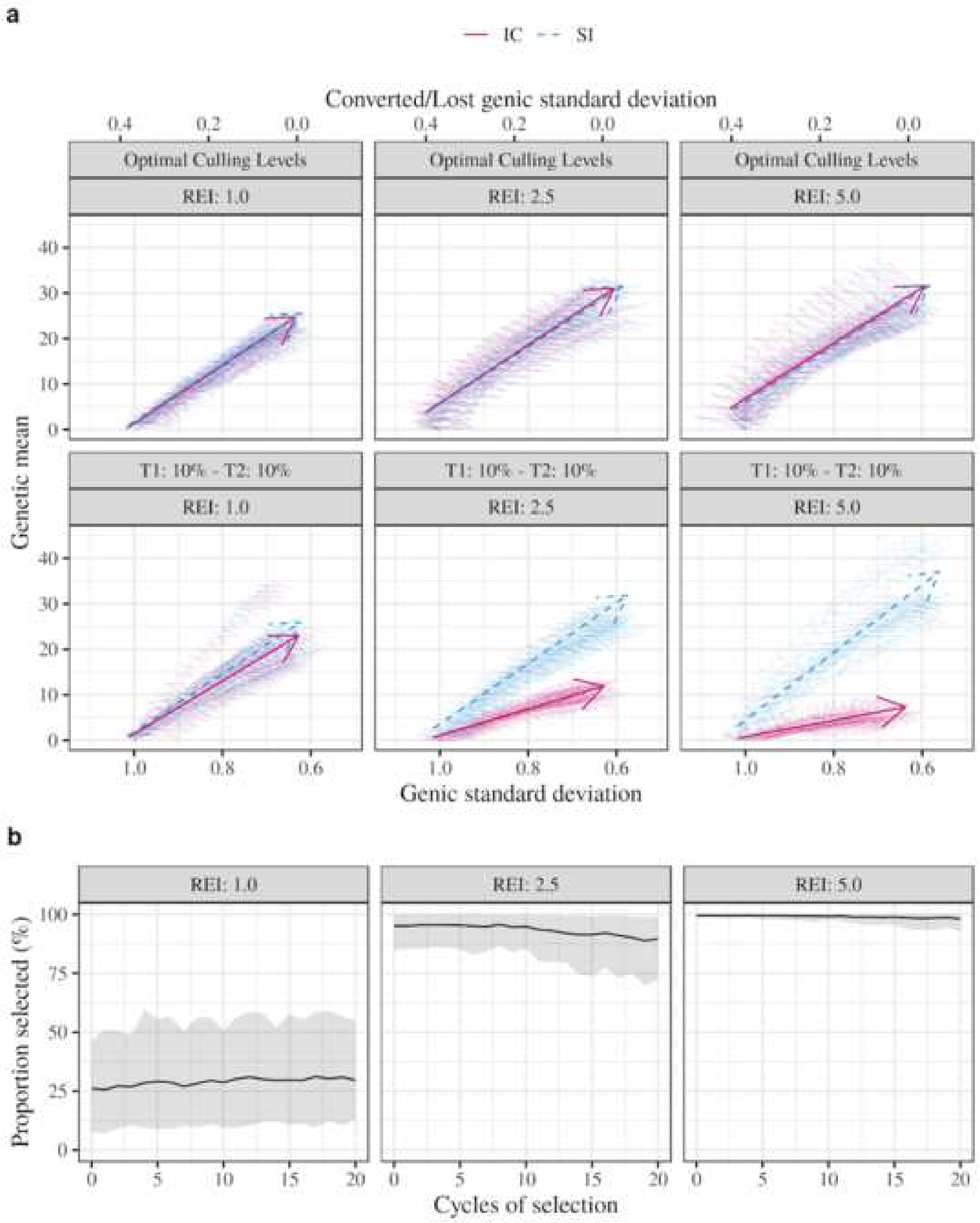

Supporting Information

Click here to access/download
Supporting Information
SupplementaryMat1.docx
