# Supplementary material for: Long-term comparison between index selection and optimal independent culling in plant breeding programs with genomic prediction
Source: PLoS One. 2021 May 10;16(5):e0235554. doi: 10.1371/journal.pone.0235554 (PMC8109766; doi:10.1371/journal.pone.0235554)
Supplement: Attachment — Submitted filename: Response to reviewers.docx [file pone.0235554.s004.docx]

**Thank you for these comments. We feel that they have improved the manuscript.**

**We have dealt with each of the comments. In what follows we describe our response and give a line number to each change made in the manuscript. For ease of review these changes are also highlighted in yellow in the resubmitted manuscript.**

**Reviewer #1: The study idea is good. However, there are some points in the text that should be better described or explained. They are all highlighted in the text. My main objection to the study is to have considered/simulated a very specific selection condition for such a broad objective. In my opinion, the simulated condition responds only to one condition, which involves negatively correlated traits. The correlation between traits is not always negative, nor is it caused by pleiotropy. Thus, it cannot be extrapolated to the whole as the study intends. I suggest that the title, objective and discussion be adjusted to a more restricted selection situation and not as wide as the study contemplated.**

***Response:*** The reviewer has a fair point. In the beginning of the development of the study we also simulated scenarios with favourably correlated traits. However, the results showed the same patterns observed for unfavourably correlated traits, only less pronounced, as there were more coincidences in the individuals being selected by the two methods (which is expected when traits are favourably correlated). We chose to keep the results that made more evident the differences between methods. Besides that, unfavourable genetic correlations are ubiquitous in breeding programs and are the main challenge faced by breeders deploying multi-trait selection. Both in our introduction (L36) and discussion (L385-387) we provide evidence of unfavourable genetic correlations between traits occurring in several economically important crops.

Given that the scenarios investigated in this study are relevant to the great majority of breeding programs, we consider our title to be appropriate even if we do not show results for other specific scenarios. We have however added clarifications to the introduction (L93) and to the discussion (L379-382) and also added a figure with results from a scenario with favourably correlated traits to our supplementary material.

**L55 -** **Reviewer comment:**

**“But in traditional breeding, individuals without information are penalized and may not be selected, correct?**

**Is your claim in the context of genomic selection? I suggest detailing more!”**

***Response:*** This claim was made in the context of phenotypic selection. The purpose of the sentence is to compare independent culling to index selection. Index selection requires phenotypes for all traits to be available, whereas with independent culling selection can be made one trait at a time. To make this distinction clearer, we have removed “for all individuals” from the sentence (L55)

**L67: Reviewer comment (highlighted the word “render”):“to better?”**

***Response:*** We have changed the word “render” to “result in” (L67)

**L110-112: Reviewer comment: “Measures with based on any study?”**

***Response:*** The reviewer has a fair point.

These are AlphaSimR default values for the species “wheat”. Given that our goal was to simulate a hypothetical crop species, we considered realistic to use values already observed for a commercial crop such as wheat. We made that explicit and also included the reference:

L111-118

L119-120: Reviewer comment

**“It was not clear to me why the traits were treated as pleiotropic.**

**This may have generated negative correlations between traits over the generations. But it is not a situation that can be generalized.**

**Needs explanations!”**

***Response:*** The reviewer has a valid point. AlphaSimR only simulates pleiotropic traits, but we believe our results can still be generalized for quantitative traits.

In our simulations we have 10,000 causal loci, each with a specific additive allele effect for each simulated trait. These allele effects are randomly assigned, resulting in loci having many possible combinations of additive effects for each trait. The loci can be assigned additive effects with the same sign (positive or negative) for both traits, they can be assigned additive effects with opposite signs for the traits, and they can be assigned additive effects with varying absolute values for each trait (i.e. a high effect for one trait and a low effect for the other trait). These are all realistic assumptions under the infinitesimal model, where both traits are controlled by an infinite number of loci with different additive effects for each trait. It also means that although QTL are pleiotropic, they are implicitly acting as non-pleiotropic (i.e., genetic correlations will also be generated by negative linkage disequilibrium). Mechanistically this is the only way to our knowledge to simulate genetic correlations in a controlled way.

L143: Reviewer comment

**“Were these F1s obtained from the 50 founder genotypes? To explain better! To account for this number of descendants, heterozygous parents were probably considered”**

***Response:*** The founder genotypes served as the initial parents in the burn-in phase and generated the first F1 population. Genotypes were then selected from the F1 population to be used as parents in the subsequent cycle. We have now modified the sentence to make this clearer.

L143-146

**L154: Reviewer comment**

**“It was not clear to me why this correlation was considered negative. The simulation refers to a specific selection situation and not what occurs in general.”**

***Response:*** This is described in lines 165-167. Our intention was to simulate a challenging scenario for the breeder. Unfavourable genetic correlations can be either positive or negative, depending on the direction of the selection being carried for each trait. In our simulations we were selecting for higher genetic values for both traits, hence we used a negative genetic correlation as unfavourable. A positive genetic correlation would be unfavourable if the direction of selection was different between traits.

Moreover, for favourably correlated traits, both methods perform very similarly, because most of the individuals selected by index selection would also be selected by independent culling. We considered that scenarios with favourable genetic correlation would not be of much additional value to the manuscript and we decided to report and discuss only results from simulations which represent challenging scenarios for multi trait selection, and which would differentiate the two methods of selection being evaluated. We have added clarifications to the introduction (L93), results (L219-222 and 228-229) and to the discussion (L379-382) and also added a figure with results from a scenario with favourably correlated traits to our supplementary material.

**L167-168: Reviewer comment:**

**“This has a consequence for genetic diversity. This does not reflect reality”**

***Response:*** The reviewer is right. Our goal in the paper was to compare index selection to the best possible results that can be obtained with independent culling, hence the optimal was chosen. Nevertheless, we also report and discuss the results (Fig. 4) for independent culling when a suboptimal proportion selected was used for each trait. The results are discussed in terms of efficiency in converting genetic diversity into genetic gain.

**L240-243: Reviewer comment**

**“Probably linked to the maximization of genetic gains that promoted a more drastic reduction”**

***Response:*** As stated in lines 407-409 of our discussion, after comparing the genetic standard deviation with the genic standard deviation, we believe this difference between the selection methods stem from the how these methods are inducing and exploiting linkage disequilibrium between the causal variants.

**L336-339: Reviewer comment**

“It is a very specific situation. What if the correlation was positive? I would like to see this situation.”

***Response:*** For favourably correlated traits, both methods would perform very similarly, because most of the individuals selected by index selection would also be selected by independent culling.

We have however added clarifications to the discussion (L379-382) and also added a figure with results from a scenario with favourably correlated traits to our supplementary material.

**L394-396: Reviewer comment**

**“I agree. However, in practice I do not know of a breeding program for clonally propagated species conducted over many cycles. I believe it is impractical.”**

***Response:*** The reviewer has a point. Conventional breeding programs for clonally propagated crops take several years for development of cultivars, which are generally used as parents in the next breeding cycle.

As shown by Gaynor et al. (2017), genomic selection allows breeding programs to be restructured by decoupling their cultivar development component from their population improvement component. As the population improvement component operates independently, this enables rapid recurrent selection with potentially several cycles of selection per year to be carried. In this context, the loss of genetic diversity needs to be carefully managed.

**L446-447: Reviewer comment**

**“economics weights of the traits?”**

***Response:*** The reviewer is correct. We have modified the sentence:

L511-512

**Reviewer #2: Review for: An economic selection index should be used instead of independent culling in plant breeding programs with genomic selection**

**This paper compares two different selection strategies, namely, independent culling and index selection in the context of multi-trait genomic selection. Simulations have been performed for two negatively correlated traits over 20 cycles of breeding for 9 different scenarios with varying selection accuracy and relative economic importance. Results demonstrate, given the economic importance of each trait, maximum genetic gains are more easily achieved with index selection. This study does not provide any new approach or methodology in terms of multi-trait selection and assumes the accurate knowledge of economic importance of traits. However, the reviewer believes it can be useful to the practitioners of genomic selection.**

**The manuscript is generally well written, and the discussions seem sound. There are some typos throughout the paper which would benefit from proofreading. The figures are not very clear, the authors should provide figures with a higher resolution.**

***Response:*** We thank the reviewer for the comments. We have proofread the manuscript again and found a number of typos. We apologise for their presence in the earlier version. Regarding the submitted figures, we used the highest resolution allowed in the Journal submission guidelines.

**Here are a few specific comments, suggestions and discussion points:**

**L93-94: What if we select for both traits simultaneously?**

***Response:*** Independent culling consists of selection for each trait being carried independently of the other traits, even if selection for multiple traits is carried simultaneously. The genotypes being selected would have been the same, although cycle time would be shorter.

**L142: It would be worthwhile to consider nonlinear selection indices as well.**

***Response:*** The reviewer has a good point and our group is planning subsequent research using non-linear indexes as well. In this study, we decided to only consider linear selection indexes. We address this in L489-491 of our manuscript, in the discussion section.

**L143-147: How are the selected parents mated? Does AlphaSimR have any strategies for mating?**

***Response:*** In this study the parents were randomly crossed. AlphaSimR also allows user-supplied crossing plans.

**L153: Report the correlation value between two traits.**

***Response:*** The reviewer is correct. We have added this information in L167-168.

**L155, Table 1: What are the optimum values? These values should be reported. Figure 4 has demonstrated the proportion selected for different relative economic importance, but it would be better to report these variables earlier when showing the results in Table 1.**

***Response:*** Table 1 is not in the results section so for this reason we excluded the results there. The optimal values of proportion selected varied across different replicates of the simulations, which is shown by the confidence margins in Figure 4. We have now provided the mean values of proportion selected for each cycle in Table S1.1 of our supplementary material in addition to the figure.

**L203, Figure 1: This Figure demonstrates the genetic values for the selected parents and the F1 population for both traits in the 3rd cycle. It will be interesting to see the same Figure for the final generation (the 20th cycle).**

***Response:*** The purpose of this figure is to illustrate which individuals were being selected by each of the methods. The characteristic pattern of individuals being selected by each method does not change over the cycles of selection. We have added the figure for the 20^th^ cycle to the supplementary results and included a sentence summary of the similarities and differences between the 1^st^ and the 20^th^ cycles on lines L219-223.

**L220, Figure 2: For 0.99 accuracy, the correlation has decreased in the first cycle when using independent culling. How do you describe/interpret that?**

***Response:*** Very good question. In the burn-in phase we used independent culling as the selection method. Hence, for the independent culling scenarios the first cycle being reported is merely a continuation of the burn-in phase, i.e. there’s nothing specific to the first cycle which differentiates it from the other cycles of selection. Given that the observed change in genetic correlation was not significant, we believe the increase in genetic correlation was not a relevant occurrence. However, we have added a sentence to report this in our results:

Line 238-239

L299: The proportion of selected parents for REI=5 is 99%. I wonder what happens if we have a large REI, say 20.

***Response:*** In this case, results reported in Hazel and Lush (1942) indicate that no selection at all would be carried for the trait with lower economic importance.

Data availability: Is the simulated data available? I couldn’t find any source links to that.

***Response:*** The reviewer has a valid point. To address this, we decided to make the code we used for the simulations available as supplementary material.
