# Supplementary material for: Long-term comparison between index selection and optimal independent culling in plant breeding programs with genomic prediction
Source: PLoS One. 2021 May 10;16(5):e0235554. doi: 10.1371/journal.pone.0235554 (PMC8109766; doi:10.1371/journal.pone.0235554)
Supplement: Attachment — Submitted filename: Response to reviewers.docx [file pone.0235554.s005.docx]

**Response to Academic Editor**

Editor comment:

*“As you can see, both of the original reviewers were satisfied with your responses to their points. Your manuscript has been passed to me to handle, and I request some clarification and changes.”*

Response: We’d like to thank the Editor for taking over the review process of the manuscript. We appreciate that the review process is now moving forward in the fairest way possible.

Throughout this rebuttal letter we will use the line numbers in the “Manuscript.docx” file. Line numbers are indicated by the letter “L” followed by the line number.

Editor comment:

*“The main point made in this paper appears to be that independent culling (IC) faces the problem of determination of selection intensity according to the economic weighting of the traits under selection. However, for maximum efficiency of genetic gain, index selection (IS) also requires estimation of these economic weights. I have read and reread the MS and cannot find any admission that when these weights are accurately estimated, the only differences between IC and IS are that IC can be applied sequentially according to when traits are scorable, and that IS allows selection of genotypes with sub-threshold scores for some traits when the remaining traits have superior scores. The authors cite both advantages as having been well known before the study was undertaken.*

*On line 143 appears “The index trait was the sum of the estimated breeding values for each trait weighted by their economic importance.” It would be helpful to see the mathematical expression for this weighting.”*

Response: We agree with the Editor and have included the formula in matrix notation in the manuscript (L142 – L146)

Editor comment:

*“But if we can develop an economic selection index based on known economic weights, why could we not perform IC based on the same weights? The authors say on line 195 “Index selection performed better than independent culling in scenarios where independent culling levels were suboptimal.” Well, sure. You could just as well say that IC performed better than IS in scenarios where IS economic weights were suboptimal. But if you know how to weight one method, you know how to weight the other. I ask the authors to address this difficulty.”*

Response: The Editor has a valid point and we modified the discussion in order to address his comments.

L414 – L419: we make more explicit in our discussion that both independent culling and index selection could lead to optimal genetic gain. However, we also considered important to emphasize that finding optimal culling levels is a much more complex task than using an index (see references 11,38,39,40,41,42 and 43). Therefore, index selection should be preferred.

Editor comment:

*“There are many writing and some organizational errors in the text; many of them are listed below with corrections. The line numbers are those of the authors’ revised submission, PONE-D-20-04603_R1.pdf. The authors are not required to adopt the exact substitute wording shown with each item, but it is strongly recommended.”*

Response:

We thank the Editor for his thorough review and for pointing out the many errors we have overlooked. We have modified the manuscript in accordance with all of the suggestions. Some of the suggestions are presented below followed by a response.

144 [You mention economic importance here, but you have not described how economic importance was assigned.]

Response: The description requested can be found in L166 – L168.

343 - 348 This material is not discussion. It is introduction and belongs only there, as part of the motivation for the study. In Discussion we discuss only the results of the present study and ideas that were first suggested by the results.

Response: The Editor has a valid point and we modified the paragraph in order to keep only what is relevant for the discussion of the results (L344 – L351).

433 This [what is “this”?]

Response: The sentences were modified in order to be more specific (L433)
