# Supplementary material for: Long-term comparison between index selection and optimal independent culling in plant breeding programs with genomic prediction
Source: PLoS One. 2021 May 10;16(5):e0235554. doi: 10.1371/journal.pone.0235554 (PMC8109766; doi:10.1371/journal.pone.0235554)
Supplement: Attachment — Submitted filename: Response to reviewers.docx [file pone.0235554.s006.docx]

Response to Academic Editor

Editor Comment:

“*Rather than ask further reviewers to read a submission I find confusing, I have chosen to try to clarify it myself. I am sorry to keep pointing out things I consider problematic, but every time I read the revised MS I find more problems and less substance. I hope these remarks will help the authors in communicating what they did and in separating what this study really established from what they could have written without ever having done a study. I can’t yet distinguish a failure to communicate from a failure of experimental rigor, but both are fatal. PLoS One standards will not prevent publication of a study of light weight, so long as it was done with rigor, and I would add: that readers can tell what it actually contributes to the field, however little that may be.”*

Response: We appreciate and we are thankful for the provided input. We agree that a failure to communicate is aggravating, hence we modified the manuscript in order to address the issues pointed out in the revision.

Editor Comment:

*“In short: what did you find out that you didn’t already know, and couldn’t have predicted from prior knowledge?”*

Response: Our study has fully detailed the effect of different methods of selection in key population parameters such as genetic diversity, genetic correlation and genetic gain over several cycles of selection. No other study has compared index selection and independent culling for that many cycles of selection. Some of the results we observed were expected and consistent with previous literature, some of the results were somewhat surprising, like the increasingly unfavourable genetic correlation between traits (which the Bulmer effect eventually helped us understand).

Editor Comment:

*“That most of your Discussion is just introduction and is independent of your experimental results is troublesome.”*

Response: We thank the editor for pointing this out, and we have moved some of the paragraphs from the Discussion to the introduction.

These paragraphs arose as a result from many discussions with other researchers and plant breeders while presenting our results. We wrongly decided to include them as a part of our discussion. However, not with the intent of claiming these findings to our study, but as important takeaway messages to breeders.

Editor Comment:

*“I ask the authors to define “accuracy” as computed in their simulation study, and to remove from Discussion all material that was not first suggested by the results (details below).I ask them to clarify how selection was performed under independent culling (details below). I ask them to acknowledge, if they agree, that all they have shown about the non-optimality of independent culling is that a flat 10% selection intensity is not as effective (by their chosen criteria) as index selection.*

*164 and Table 1 Four levels of accuracy... => You have nowhere defined accuracy or explained how these levels were applied to the simulation. You could presumably show this mathematically or, failing that, algorithmically. Even up to line 201, citing “the accuracy of selection”, you have not defined accuracy. The paper simply makes no sense without such a definition.”*

Response: We defined accuracy as the correlation between true and estimated breeding values. This definition is present in the manuscript. We now slightly modified the sentence to highlight it better (L138-L139)

Editor Comment:

*“171-172 Maximised the genetic gain [to establish culling levels]? The true genetic gain is not known to a breeder. If you are using that, the simulation is not realistic. This is important, because your conclusions cite “optimal” culling levels. Did you maximise the genetic gain estimated from the phenotype, a parameter that would be available to a breeder? But then in line 176-177 you write that you did not use genetic gain at all, but economic value, and applied index selection to determine culling levels. Supplementary figure S.1.1 shows that this paragraph mixes the two methods without distinction. But the main MS should not depend on the supplementary material for explanation.”*

Response: We have included in the manuscript that optimal independent culling aimed at maximizing the genetic gain for the index trait, but it does it by selecting on one trait at a time. We obtained the index trati from estimated breeding values of the traits (L180-L181). We thank the Editor for pointing that out.

The figure in S.1.1 only represents the selection methods graphically, it doesn’t add information that is not already included in the Material and Methods.

1) In the independent culling method selection is performed one trait at a time as is often done in plant breeding. Individuals are ranked based on one trait and the individuals with the highest values are kept while the other individuals are culled. The individuals that were kept are then ranked based on a second trait and then culled again. This process is repeated for each additional trait under selection.
2) In the selection index method individuals are selected for all traits at once, generally by using a linear combination of the traits, which is the index. Individuals are ranked based on the index and the ones with the highest values are kept.

Hence, in the optimal independent culling methodology we do not mix the two methods. The index trait is used as a reference for establishing which are the optimal selection intensities we need to use for each trait. Once we have these selection intensities (i.e. the number of individuals we need to cull in each stage) independent culling is carried as described in 1. We did not use the index trait to rank the individuals, selection was carried out for each trait at a time.

Editor Comment:

*“198 independent culling levels were suboptimal => This is deceptive, because you did not simulate “suboptimal levels”. You simulated only one, a 10% selection proportion.”*

Response: We agree with the Editor and we have modified the sentence accordingly (L207-208)

Editor Comment:

*“313 Accurately assessing the economic importance of the traits is essential => Where is the experimental evidence for this assertion, given that you have not defined accuracy or, as far as your M&M indicates, simulated any process by which a breeder might assess economic importance? Is the 10% selection variant the only test you are using to represent “inaccurate” estimation of economic importance?*

*412 Same as 313. How can you make this assertion?”*

Response: We agree with the Editor and we have removed the sentences from our manuscript

Editor Comment:

*“402-407, 414-419; this was all known before the study and is not discussion of the results.*

Response: Please see below.

*412-413. You did not test estimation errors; you compared only the flat 10% (which no sensible breeder would be likely to apply for traits with clear differences in economic importance) with projection of genetic gain.*

Response: Please see below.

*421-428 This material is not discussion of your results and is not a conclusion from your results. You knew it from the literature before the study was performed. The same is true of 435-441; you could have written it without doing any experiment. Your simulation doesn’t include any test of estimation errors of culling levels.*

Response: Please see below.

*429-434 All except the second sentence are introduction. They serve as background knowledge and motivation for the decision to perform this study. The second sentence just repeats a claimed finding; repetition doesn’t add evidence.*

Response: Please see below.

*435-452 is pure introduction. It is not derived from any findings of this study.*

Response: Please see below.

*463-466 is introduction. That optimal culling levels are complex to estimate was given, not shown in the study.*

*As a reminder: an Introduction poses the research problem and says what we know about it, what we wish to find out, and how we propose to find it out.*

*The M&M and Results section seem to be in order in this MS, except for the omission in explaining “accuracy” and how it was simulated.*

*A Discussion explains and draws inferences from the results. Unlike the Introduction, it’s concerned with presenting ideas that we didn’t already know and that were first suggested by the results. If there is little to discuss, the section should be short. Introductory material, such as composes much of the Discussion in this MS, should be moved to the Introduction, unless it is mere repetition of material that is already there.”*

Response: We agree with the Editor and we have considered all these comments carefully in order to modify the manuscript. Some of the paragraphs in our discussion were excluded from the manuscript and some were moved to the introduction, in accordance with the remarks.

We thank the Editor’s for these comments, and we belie this revision helped us improve our discussion. We also changed our title in accordance to the comments.

The flat 10% selection intensity for both traits would have been the breeder’s intuitive choice in scenarios with a relative economic importance of 1.0 (traits with equal economic importance). Even in that case, optimal culling levels performed better than the intuitive choice. We have decided to argue in our discussion that this result indicates that culling levels should not be chosen based on intuition and should be estimated accurately aiming at maximizing economic value.

We have kept the remark about the complexity of estimating optimal culling levels in the discussion, but we removed it from our conclusions. Since we showed that both optimal independent culling and index selection lead to equivalent genetic gains, the complexity is an important caveat when deciding between the two methods.
